# In Situ Functionalization of Polar Polythiophene-Based Organic Electrochemical Transistor to Interface In Vitro Models

**DOI:** 10.1021/acsami.4c09197

**Published:** 2024-09-27

**Authors:** Sebastian Buchmann, Pepijn Stoop, Kim Roekevisch, Saumey Jain, Renee Kroon, Christian Müller, Mahiar M. Hamedi, Erica Zeglio, Anna Herland

**Affiliations:** † Division of Nanobiotechnology, Department of Protein Science, SciLifeLab, KTH Royal Institute of Technology, Stockholm 177 65, Sweden; ‡ AIMES—Center for the Advancement of Integrated Medical and Engineering Sciences at Karolinska Institutet and KTH Royal Institute of Technology, Stockholm 171 65, Sweden; § Department of Neuroscience, Karolinska Institutet, Stockholm 171 77, Sweden; ∥ Division of Micro and Nano Systems, Department of Intelligent Systems, KTH Royal Institute of Technology, Stockholm 100 44, Sweden; ⊥ Department of Science and Technology, Laboratory of Organic Electronics, Linköping University, Norrköping 602 21, Sweden; # Department of Chemistry and Chemical Engineering, Chalmers University of Technology, Gothenburg 412 96, Sweden; ¶ Division of Fibre Technology, Department of Fibre and Polymer Technology, KTH Royal Institute of Technology, Stockholm 100 44, Sweden; ∇ Digital Futures, Stockholm 100 44, Sweden; ○ Wallenberg Initiative Materials Science for Sustainability, Department of Materials and Environmental Chemistry, Stockholm University, Stockholm 106 91, Sweden

**Keywords:** OECTs, OMIECS, functionalized conjugated polymer, in situ functionalization, bio interface, cell barrier, Caco-2

## Abstract

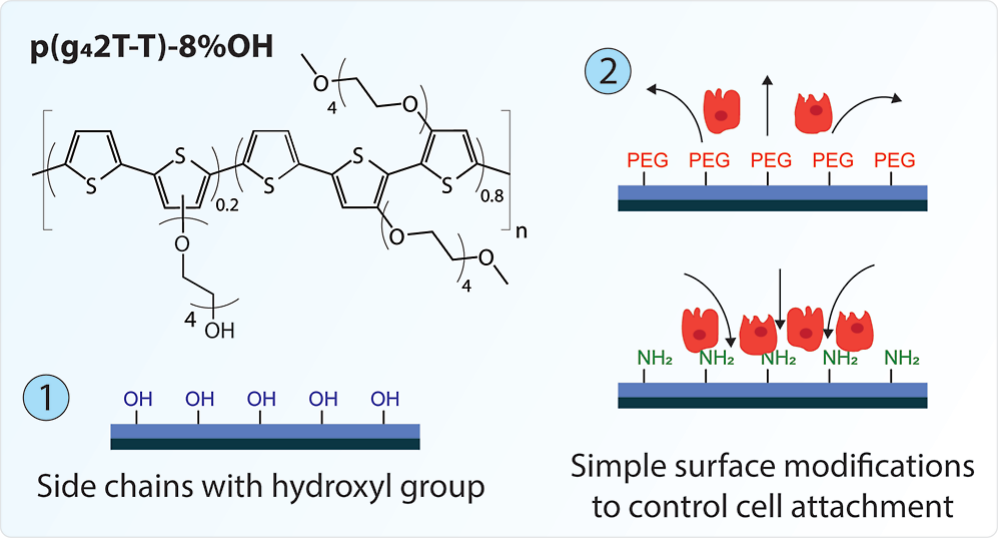

Organic mixed ionic-electronic conductors are promising materials for interfacing and monitoring biological systems, with the aim of overcoming current challenges based on the mismatch between biological materials and convectional inorganic conductors. The conjugated polymer/polyelectrolyte complex poly(3,4-ethylenedioxythiophene):polystyrenesulfonate (PEDOT/PSS) is, up to date, the most widely used polymer for in vitro or in vivo measurements in the field of organic bioelectronics. However, PEDOT/PSS organic electrochemical transistors (OECTs) are limited by depletion mode operation and lack chemical groups that enable synthetic modifications for biointerfacing. Recently introduced thiophene-based polymers with oligoether side chains can operate in accumulation mode, and their chemical structure can be tuned during synthesis, for example, by the introduction of hydroxylated side chains. Here, we introduce a new thiophene-based conjugated polymer, p(g_4_2T-T)-8% OH, where 8% of the glycol side chains are functionalized with a hydroxyl group. We report for the first time the compatibility of conjugated polymers containing ethylene glycol side chains in direct contact with cells. The additional hydroxyl group allows covalent modification of the surface of polymer films, enabling fine-tuning of the surface interaction properties of p(g_4_2T-T)-8% OH with biological materials, either hindering or promoting cell adhesion. We further use p(g_4_2T-T)-8% OH to fabricate the OECTs and demonstrate for the first time the monitoring of epithelial barrier formation of Caco-2 cells in vitro using accumulation mode OECTs. The conjugated polymer p(g_4_2T-T)-8% OH allows organic-electronic-based materials to be easily modified and optimized to interface and monitor biological systems.

## 1 Introduction

The interface between biological systems and electronic devices determines the ability to monitor and investigate physiological processes. Organic mixed ionic-electronic conductors (OMIECs) have great potential to interface and interact with biological systems both in vitro and in vivo.^1,2^ In contrast to conventional metals and inorganic conductors and semiconductors, OMIECs provide mixed ionic and electronic conductivity^3^ while also having side chains where chemical modifications allow fine-tuning of electronic properties or controlling compatibility with cells and tissues.^4^ OMIECs can also be processed by a variety of cost- and time-effective patterning techniques, such as printing,^5,6^ spray/spin-coating,^7,8^ gel filtration-based micromachining,^9^ and direct laser writing.^10^

OMIECs comprise mostly conjugated polymers. Among other possible applications to fabricate ion pumps,^11^ supercapacitors,^12^ batteries,^13^ or electrochromic displays,^14^ OMIECs are used as active materials to fabricate organic electrochemical transistors (OECTs): a three-terminal device that makes use of the mixed ionic/electronic conductivity to transduce and amplify small ionic signals. In the classical OECT configuration, the OMIEC channel material connects the source and drain electrodes, and a gate electrode is connected to the channel through an electrolyte solution.^15^ Ions injected into the channel, upon application of a gate voltage or local changes in ionic concentration, modify the doping of the channel material, thereby altering its conductivity. The volumetric penetration of ions into the bulk of the channel material leads to relatively higher signal amplification (transconductance) compared to other transistor configurations, such as electrolyte-gated transistors, that just rely on changes in areal capacitance.^15,16^ Because of this amplification property and operation at low voltages, OECTs have been used for a wide range of bioelectronic applications, including monitoring changes in ion fluxes produced by electrophysiologically active cells and tissues (such as neural or cardiac) in vitro or in vivo.^17−21^

For nonelectroactive cells (e.g., epithelial cells), previous studies showed that the steady-state performance of OECTs is sensitive to changes in surface charge and morphology of cells cultured on top of the active material, making it possible to monitor the activity of adherent cells.^22^ Furthermore, the switching response time of an OECT has been used to monitor cell coverage and barrier formation.^23−28^ Barrier-forming cells create distinct separations between different physiological environments. Intestinal epithelial cells, for example, separate the human body from microbial infections and mediate the absorption of nutrients.^29^ Monitoring the barrier integrity of intestinal epithelial cells in vitro can give important insights for toxicological studies,^30^ drug delivery,^31^ and disease modeling.^26,32,33^ A tight cell barrier on top of the OECT channel hinders ion movement into the polymer film and reduces the switching response times, enabling even the discrimination between different cell types based on their barrier characteristics to study invasion and migration of carcinoma cells.^27^ Moreover, by varying the dimensions of the channel area, it is possible to optimize the measuring conditions for cells that form a tight or leaky cell barrier.^26^

When fabricating an OECT, the OMIEC material largely influences the device’s operation and performance. The examples mentioned above where OECTs were used to monitor cell barrier integrity were all performed using the conjugated polymer/polyelectrolyte complex poly(3,4-ethylenedioxythiophene)/poly(styrenesulfonate) (PEDOT/PSS). While PEDOT/PSS has the advantage of being commercially available and is considered a benchmark material due to the high conductivity, its main disadvantages are that (1) it is challenging to modify PEDOT/PSS from a synthetic point of view. There are examples of introducing functional groups through electro-polymerizing EDOT variants^34,35^ or via plasma treatment of PEDOT/PSS surface.^36,37^ However, electropolymerization suffers from limited scalability, while plasma treatment reduces the conductivity^37^ and thickness^36^ of PEDOT/PSS, therefore compromising the final device performance. (2) OECTs using PEDOT/PSS operate in depletion mode where high on/off rations can only be achieved when the applied gate voltage bias switches from its maximum negative to maximum positive applicable value. In most reported pulse measurement experiments where barrier integrity has been evaluated, the gate voltage was switched from 0 V into the positive regime, leading to a significantly lower on/off ratio.^38^ Additionally, as a negative gate voltage is applied to turn a PEDOT/PSS-based accumulation mode OECTs off, it is possible to study the transport of cations through a cell layer or a lipid bilayer but not the transport of anions.^39^

Recent work with polar polythiophenes such as p(g_4_T2-T) or p(g_4_T2-TT) with ethylene glycol (EG) side chains has reported superior OECT performance, benchmarked as the product of mobility and volumetric capacitance μC*, providing the possibility to introduce and test a variety of different side chains and operate in accumulation mode.^40−45^ However, EG chains are known to have antifouling properties.^46,47^ While this can be beneficial for specific medical device applications, it is not preferable for in vitro sensing, as cells are not expected to adhere well to surfaces with exposed EG chains. Indeed, the only report on in vitro cytotoxicity of conjugated polymers with EG side chains was performed without direct contact of the cells (fibroblasts) with the conjugated polymer film.^48^ A conjugated polymer that can be tuned to improve and prevent interaction with cells could open up new possibilities for in vitro and in vivo monitoring.

**Figure 1:**
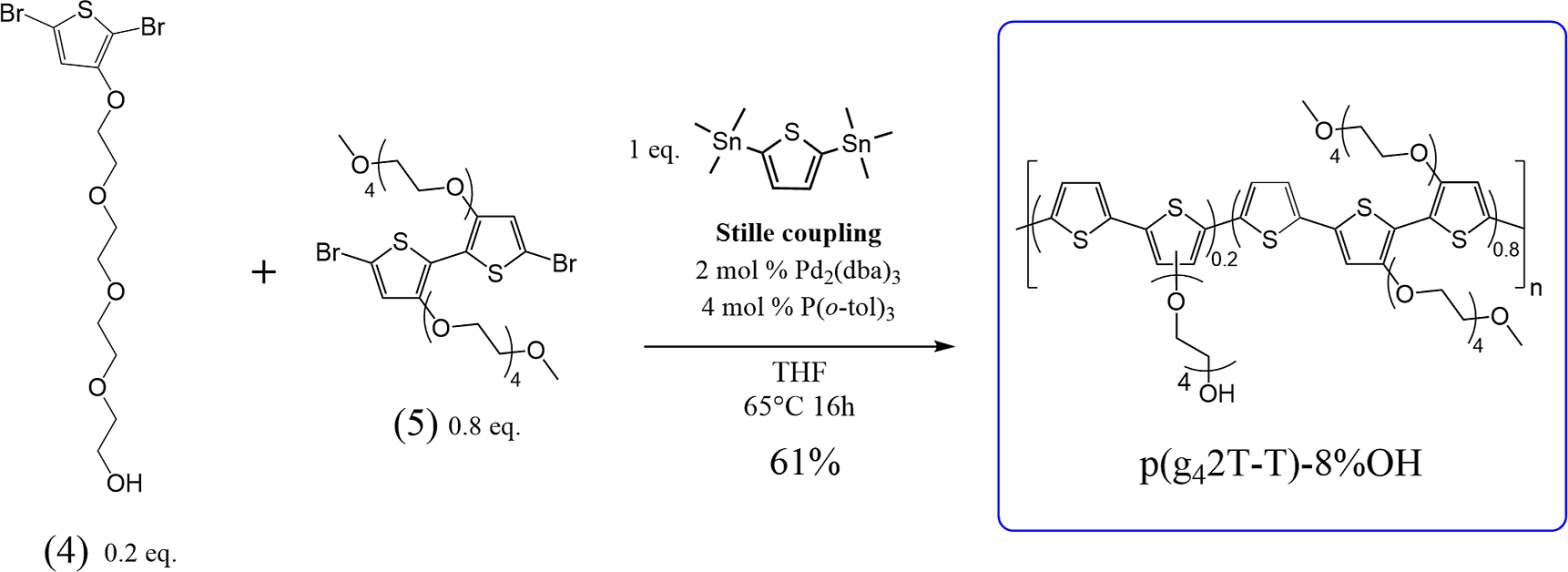
Reaction scheme showing the polymerization reaction of conjugated polymer p(g_4_2T-T)-8% OH, where 8% of the side chains are functionalized with a hydroxyl group.

Here, we address this by introducing a new polar polythiophene-based polymer p(g_4_2T-T)-8% OH, where 8% of the glycol side chains are functionalized with hydroxyl groups (see Figure 1). The newly introduced functional groups allow simple chemical modifications on the polymer film surface, such as via silanization. We showed that by modifying the surface of p(g_4_2T-T)-8% OH with two silanes: (3-aminopropyl) trimethoxysilane (APTMS) and silane-poly(EG)-acetic acid (silane PEG-COOH), it is possible to improve or hinder the growth of barrier-forming human colon carcinoma (Caco-2) cells or differentiating Lund Human Mesencephalic (LUHMES) neuronal cells. We could further show that p(g_4_2T-T)-8% OH can be used to fabricate functional accumulation mode OECTs despite surface functionalization. Finally, we designed a simple OECT cell culture device and used it to monitor the cell barrier formation of Caco-2 cells, showcasing how p(g_4_2T-T)-8% OH-based OECTs can be used to monitor in vitro models.

## 2 Materials and Methods

### 2.1 Materials

Chloroform (product no. 366927), 1,2-dichlorobenzene (product no. 240664), 4-bromoanisole (product no. B56501), poly l-ornithine hydrobromide (PLO, product no. P3655), human plasma fibronectin (product no. FC010), tetracycline (product no. T7660), goat serum (product no. G9023), and antimouse IgG1 CF555 and APTMS (product no. 281778) were purchased from Merck. Silane PEG-COOH 2 kDa (product no. PG2-CASL-2k) was purchased from Chemotronica. Acetone (product no. 20063.365), round coverslips (13 mm, product no. 631-0150), and ethanol (product no. 20821.365) were purchased from VWR. Polyimide precursor solution (polyamic acid in an *N*-methyl-2-pyrrolidone, product no. PI2545) was purchased from HD MicroSystems. Caco-2 cells (clone C2BBe1, product no. CRL-2102) were purchased from ATCC. DMEM (high glucose, GlutaMax Supplement, pyruvate, product no. 10569010), insulin-transferrin-selenium (ITS-G, product no. 41400045), Dulbecco’s phosphate-buffered saline (DPBS, product no. 14190094), PBS Tablets (product no. 18912014), advanced Dulbecco’s modified Eagle’s medium/F12 (Adv-DMEM/F12, product no. 12634010), N2 (product no. 17502-048), and fetal bovine serum (FBS, product no. A384000) were purchased from Gibco. Penicillin/streptomycin (P/S, product no. 0503) was purchased from ScienCell. Polydimethylsiloxane (PDMS, SYLGARD 184 Silicone Elastomer Kit) was purchased from Dow. l-Glutamine (product no. 25030024) was purchased from Thermo Scientific. Triton X-100 (product no. HFH10), ZO-1 antibody (product no. 339100), DAPI (product no. D1306), and Alexa Fluor 488 Phalloidin (product no. A12379) were purchased from Invitrogen. Low methanol formaldehyde (product no. 4235.4) was purchased from Roth. Recombinant human fibroblast growth factor (FGF, product no. 233-FB) was purchased from R&D Systems. Dibutyryl cAMP (product no. S7858) was purchased from Selleck Chemicals. Recombinant human GDNF (product no. 450-10) was purchased from PeproTech. All chemicals used for the synthesis of monomers and polymers were purchased from Sigma-Aldrich. All chemicals were used as received with the exception of 2,5-bis(trimethylstannyl)thiophene, which was recrystallized from methanol prior to use.

### 2.2 Synthesis of p(g_4_2T-T)-8% OH and p(g_4_2T-T)

The conjugated polymer p(g_4_2T-T) (number-average molecular weight *M*_n_ ≈ 24 kg/mol) was synthesized as described previously by Kroon et al.^49^ A detailed description of the individual synthesis steps for p(g_4_2T-T)-8% OH is written in Supporting Information (including Figure S1 and S11–S13), and a schematic overview of the polymerization reaction is shown in Figure 1. In brief, the synthesis of the glycol-OH-functionalized thiophene monomer was done by first monoprotecting tetraethylene glycol with *tert*-butyldimethylsilyl (TBDMS)-chloride. The product **1** was then reacted with 3-bromothiophene via Ullman aryl ether coupling, after which the obtained thiophene intermediate **2** was dibrominated with *n*-bromosuccinimide (NBS) to give compound **3**. After removal of the protecting group with tetrabutylammonium fluoride (TBAF), the glycol-functionalized thiophene monomer **4** was obtained as a light yellow, viscous oil. To obtain the OH-functionalized p(g_4_2T-T)-8% OH, the g_4_T monomer **5**, the OH-functionalized monomer **4**, and 2,5-bis(trimethylstannyl)thiophene were polymerized via Stille coupling using a 0.8:0.2:1 molar ratio. After purification, the target material was obtained as a blue solid with a number-average molecular weight of *M*_n_ ≈ 18 kg/mol (Figure S14). The presence and the content of hydroxyl functional groups (8% of all glycol side-chains bear OH-functional groups) were verified by ^1^H NMR (Figure S13). Inductively coupled plasma mass spectrometry (IPC-MS) was performed by MikroLab Kolbe, Oberhausen, Germany, using 150 mg of purified p(g_4_2T-T)-8% OH polymer to determine Pd and Sn traces originating from Stille coupling.

### 2.3 Deposition of p(g_4_2T-T)-8% OH and p(g_4_2T-T)

To deposit p(g_4_2T-T)-8% OH or p(g_4_2T-T), the conjugate polymers were dissolved at 10 mg/mL in 1:1 chloroform/1,2-dichlorobenzene with 5% (v/v) 4-bromoanisole and spin-coated on the sample substrate at 2500 rpm for 60 s. The coated samples were then annealed in the oven at 130 °C for 10 min.

### 2.4 Silanization/Surface Modification

To perform the silanization reactions, spin-coated samples were incubated overnight at room temperature in (1) 2% (v/v) APTMS, 93% (v/v) ethanol 5% (v/v) distilled water, or (2) 3 mg/mL silane PEG-COOH 2 kDa in 95% (v/v) ethanol 5% (v/v) distilled water. Silanized samples were washed three times in 95% ethanol in a water solution. Control samples were incubated in 95% ethanol (v/v) and 5% (v/v) water solution.

### 2.5 Nuclear Magnetic Resonance Spectroscopy

NMR spectra were recorded with an automated Agilent (Varian) MR 400 MHz spectrometer (equipped with “one-probe”) with CDCl_3_ as the solvent. In all cases, the peak values were calibrated relative to the residual solvent signals (CDCl_3_, 7.26 ppm).

### 2.6 Atomic Force Microscopy

The samples were characterized under the tapping mode of Bruker Dimension Icon atomic force microscopy (AFM) using a XSC11/PT silicon tip from MikroMasch (cantilever *T* = 270 nm, *L* = 210 μm, *W* = 30 μm, *f*_0_ = 80 kHz, *k* = 2.7 N/m). The spin-coated polymers on glass substrates were imaged at three different locations for each sample with a resolution of 512 pixels × 512 pixels. The images were then processed and analyzed by using Gwyddion 2.63 software. The roughness was determined using the “Statistical Parameter” tool on Gwyddion.

### 2.7 Contact Angle Measurement

Contact angles were measured with a ThetaLite optical tensiometer (TL100, Biolin Scientific) using the sessile drop technique. Measurements were performed on three different areas of each sample by dropping a 4 μL drop of deionized water.

### 2.8 OECT Fabrication

Gold electrodes were fabricated using a standard lift-off deposition procedure. The electrode layout was designed by using L-edit software, and a chrome mask fabricated from Compugraphics was used. First, a 10 nm titanium adhesion layer was deposited, followed by a 100 nm thick gold layer.

OECTs with a channel size of 200 μm × 20 μm were used to characterize the material performance. The conjugated polymer solution was deposited on the gold electrodes, as described in paragraph 2.3, using spin-coating. The active layer was patterned manually using a cotton tip soaked with acetone, and electrode contacts were insulated using cellulose acetate tape. A Tencor-P15 stylus profilometer was used to measure the thickness of the spin-coated polymer films.

OECTs with a channel size of 100 μm × 10 μm were used to measure the cell barrier formation of Caco-2 cells. The OECTs were fabricated as described previously using the direct laser patterning method.^10^ In brief, the polyimide precursor was spin-coated at 6000 rpm for 60 s on top of the gold electrodes and then baked at 250 °C for 30 min to obtain an insulating polyimide layer. A femtosecond laser (Photonics Professional GT2, Nanoscribe) was used at high power (pulse energy of 600 pJ) to pattern the insulating layer, opening up a window of 120 × 30 μm on top of the channel electrode area. The conjugated polymer solution was then deposited as described in paragraph 2.3 using spin coating, and a femtosecond laser was used at lower power (pulse energy of 300 pJ) to pattern the conjugated polymer layer and define the active channel area by outlining a 15 μm wide rectangle around the channel. To prepare the OECTs for the cell culture experiments, a PDMS well was used on top of the glass substrate to confine the area where the cells should grow. The PDMS well was obtained with the help of a 3D printed mold (SLA printer Form 3, clear ResinV4, Form laboratories).^50^ PDMS was performed using a SYLGARD 184 Silicone Elastomer Kit. Briefly, SYLGARD 184 prepolymer and the curing agent were mixed at a weight ratio of 10:1, poured into the mold, degassed, and cured at 60 °C overnight. The PDMS well was then glued on top of the OECT electrode sample using a thin layer of uncured PDMS and kept in the oven at 60 °C overnight to fully bond and obtain a tightly sealed well.

### 2.9 Cell Culture

Caco-2 cells were maintained following the recommended protocol from ATCC. In brief, Caco-2 cells were cultivated in DMEM (high glucose, glutaMAX supplement, pyruvate) supplemented with 10% FBS, 1% ITS, and 1% P/S in standard tissue culture treated flasks (T75) at 37 °C and 5% CO_2_. Cells were passaged (2000 cells/cm^2^) once a week when reaching 80–90% confluency, and media was refreshed twice a week.

For the surface coverage experiments, p(g_4_2T-T) or p(g_4_2T-T)-8% OH-coated and silanized round coverslips were sterilized in ethanol for 5 min and washed in DPBS three times. Caco-2 cells were seeded on the coverslips in conventional 24 well plates at a density of 15,000 cells/cm^2^. Media without FBS supplement was used and replaced with fresh media every 3 days.^51^

For the OECT cell barrier measuring experiments, the OECT electrode array equipped with the PDMS well was sterilized by soaking in ethanol for 5 min and washed three times with DPBS. Caco-2 cells were seeded on the OECT samples at a density of 40,000 cells/cm^2^. Media without FBS supplement was used and replaced with fresh media every 3 days. Caco-2 cell passage numbers between 55 and 62 were used for all experiments.

LUHMES cells were cultivated according to the protocol described by Scholz et al.^52^ In brief, LUHMES cells were grown in Adv-DMEM/F12 media, supplemented with 1× N2, 2 mM l-glutamine, and 40 ng/mL FGF at 37 °C and 5% CO_2_ and passaged 1:10 when reaching 80% confluency. To differentiate LUHMES cells on p(g_4_2T-T)-8% OH, cells were first seeded in tissue culture flasks at a density of 46,000 cells/cm^2^. After 24 h, the growth media was replaced by the differentiation media composed of Adv-DMEM/F12 media, supplemented with 1× N2, 2 mM l-glutamine, 1 mM dibutyryl cAMP, 1 μg/mL tetracycline, and 2 ng/mL GDNF. After 48 h, predifferentiated LUHMES cells were passaged and reseeded on p(g_4_2T-T)-8% OH sterilized glass coverslips at 150,000 cells/cm^2^ density and kept in differentiation media. P(g_4_2T-T)-8% OH substrates were coated with 5 μg/mL fibronectin solution for 4 h before seeding the LUHMES cells.

### 2.10 Electrical Measurements of the OECTs

OECT measurements to characterize the material were performed using a Keithley 4200A-SCS parameter analyzer (Tektronix) equipped with two source measurement units. Measurements were performed by using 10 mM PBS as an electrolyte and a Ag/AgCl pellet as a gate electrode.

OECT measurements to sense the Caco-2 cell barrier formations were performed using two individual source meters (2410 and 2401, Tektronix) connected via GPIB cables and controlled by using the Keithley Kickstart software (Tektronix). Cell media used to cultivate the Caco-2 cells as described in Section 2.9 was used as the electrolyte and a Ag/AgCl pellet as a gate electrode. The measurements were conducted on a heated metal plate at 37 °C in atmospheric air. Each device sample contained two OECT electrodes and was measured only once, either 1, 4, or 8 days after seeding the cells.

### 2.11 Imaging and Image Analysis

Brightfield images were taken using an inverted CKX41 Olympus microscope equipped with an AmScope MU2003-BI camera. Caco-2 cell coverage image analysis was done using Fiji software^53^ following the protocol described by Čepaepa^54^ with the following optimized parameters for the Caco-2 cells and our imaging setup:

- “Enhance Contrast···”, “saturated = 0 equalize”
- “Canny Edge Detector”, “gaussian = 1.75 low = 0.1 high = 8”
- “Maximum···”, “radius = 20”
- “Options···”, “iterations = 10 count = 3 pad do = Close”
- “Options···”, “iterations = 15 count = 3 do = Open”
- “Options···”, “iterations = 2 count = 3 pad do = Erode”

For immunocytochemistry fluorescent images, cells were fixed in 4% low methanol formaldehyde and then incubated in DPBS with 10% goat serum and 0.1% TritonX-100 for 1 h. Anti-ZO-1 (diluted 1:200) was incubated in DPBS with 1% goat serum and 0.01% Triton X-100 at 4 °C overnight, followed by antimouse CF555 for 1 h at room temperature, and lastly, DAPI (1:1000) and phalloidin 488 (165 nM) for 1 h at room temperature. Fluorescent imaging was performed using a Zeiss Cell Observer microscope equipped with an Zeiss AxioCam MRm camera.

### 2.12 Calculations and Statistics

P values of the two-sample *t* tests and one-way ANOVA tests were calculated by using OriginLab Pro software. Growth curves of Caco-2 cells were fitted with OriginLab to a sigmoidal logistic curve
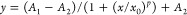
1

OECT on/off switching times of the OECT were calculated by the time it took to reach 90% or 10% of the maximum drain current, where the baseline current where 0 gate voltage was applied was subtracted and set to 0%. The transconductance *g*_m_ was obtained by taking the derivative of the transfer curve and averaging 10 points to smoothen the curve. Normalized maximum transconductance was calculated using the following equation
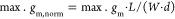
2where *L*, *W*, and *d* correspond to the length, width, and thickness of the OECT channel, respectively. The threshold voltage *V*_TH_ was obtained by extrapolating the linear regime of the *I*_DS_^0.5^ vs *V*_G_ curve. The on/off current ratio was derived from the maximum and minimum currents of the transfer curve. The product of the electronic carrier mobility μ and the volumetric capacitance C* were calculated using the following equation
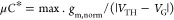
3

## 3 Results and Discussion

### 3.1 Hydroxyl-Functionalized Polar Polythiophene Polymer p(g_4_2T-T)-8% OH

The polar polythiophene-based polymer p(g_4_2T-T)-8% OH was synthesized as illustrated in the schematic overview in Figures 1 and S1 (see Materials and Method Section and Supporting Information for details). The OH-functionalized monomer **4** and the g_4_T monomer **5** were polymerized via Stille coupling. The monomer compound **4** contains the glycol side chain terminated with a hydroxyl group, introducing an additional functional group into the final polymer, which can be used for further postpolymerization reactions. Compounds **4** and **5** were combined at a molar ratio of 1:4 for the polymerization reaction to obtain a final p(g_4_2T-T)-OH polymer where 11% of the side chains theoretically bear the hydroxyl group. The ratio of 1:4 was chosen to incorporate a sufficient amount of hydroxyl groups, ensuring that postpolymerization chemistry can be performed while retaining the good electrical performance of the related p(g_4_2T-T) polymer (see Figure S2A, a polythiophene with polar tetraethylene glycol side chains obtained by polymerizing solely compound **5**).^41,49^^1^HNMR showed that 8% of all glycol side chains bear the hydroxyl functional group in the final p(g_4_2T-T)-8% OH polymer (Figure S13). IPC-MS revealed that purified p(g_4_2T-T)-8% OH contains 17 and 71 ppm of Pd and Sn traces, respectively, originating from the Stille coupling. Such low Pd and Sn traces are essential as they could otherwise form toxic complexes, making the polymer incompatible with in vitro cell culture models.^55^

P(g_4_2T-T)-8% OH displays a solubility of 10 mg/mL in both chloroform and 1,2-dichlorobenzene. However, at room temperature, p(g_4_2T-T)-8% OH dissolves faster in 1,2-dichlorobenzene. To facilitate the spin-coating deposition of p(g_4_2T-T)-8% OH, the polymer was dissolved in an optimized 1:1 mixture of chloroform and 1,2-dichlorobenzene with 5% (v/v) 4-bromoanisole. 1,2-Dichlorobenzene helped to dissolve the polymer, the fast evaporation of chloroform improved the spin-coating performance, and 4-bromoanisole was added to promote the crystallization process.^56^

### 3.2 Surface Modification on p(g_4_2T-T)-8% OH to Control Interaction with Biological Materials

**Figure 2:**
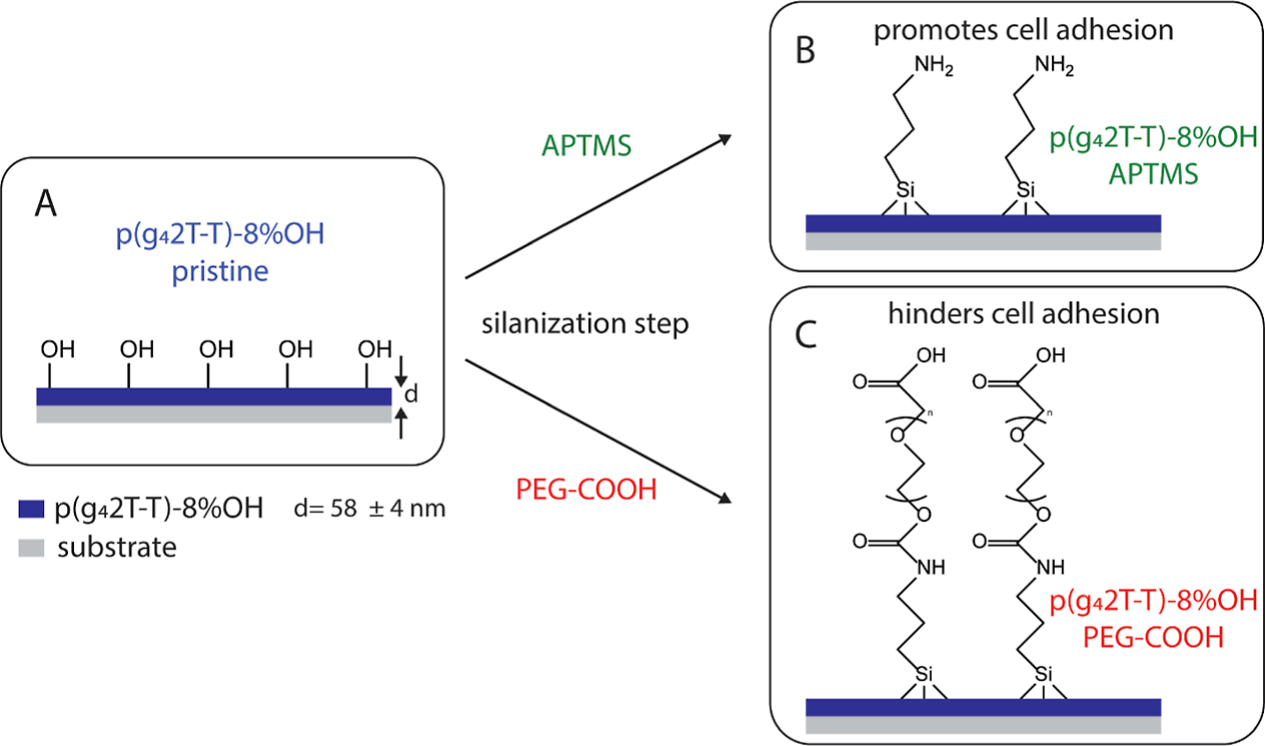
Schematic overview showing the surface modification reaction of spin-coated p(g_4_2T-T)-8% OH films. (A) Pristine p(g_4_2T-T)-8% OH containing hydroxyl groups where the silanization reactions can occur with (B) APTMS to introduce an amine group and promote cell adhesion or (C) PEG-COOH silane to introduce additional PEG chains hindering cell adhesion.

Controlling and enabling cell attachment and growth on top of the conjugated polymer film is one of the key requirements to be able to interface and monitor parameters such as coverage, activity, or barrier-forming properties.^1^ The newly introduced hydroxyl groups in p(g_4_2T-T)-8% OH allow simple silanization reactions on the spin-coated polymer surface. We tested if we could modify the surface properties of p(g_4_2T-T)-8% OH using the two different types of silanes: (1) APTMS and (2) PEG-COOH silane (see Figure 2A–C). We chose these two molecules based on the opposing properties of amino and PEG-COOH functional groups when interacting with proteins and cells. The amino groups of APTMS promote the adhesion of proteins and cells, whereas the long PEG chain of PEG-COOH prevents adhesion.^57,58^

To perform the silanization step, spin-coated p(g_4_2T-T)-8% OH samples were incubated in an ethanol-based silane solution containing APTMS (2% v/v) or PEG-COOH (3 mg/mL). Control pristine samples were incubated in ethanol for the same amount of time (see experimental Section 2.4 for details). The surface contact angle with water decreased from 73.6 ± 1° for the pristine surface to 63.9 ± 0.3 and 68.1 ± 0.1° (*n* = 6) for the surface silanized with APTMS and PEG-COOH, respectively, verifying that the surface chemistry changed (see Figure S3).

We further performed AFM measurements to gain information about the surface topography of the new p(g_4_2T-T)-8% OH polymer and its modification with APTMS or PEG-COOH. With a surface roughness value of RMS = 2.1 nm, the p(g_4_2T-T)-8% OH surface is homogeneously patterned (see Figure S2C, left). The surface topography and roughness are comparable to the surface of the related p(g_4_2T-T) polymer (RMS = 2.0 nm, see Figure S2B), indicating that the partly hydroxylated side chains of p(g_4_2T-T)-8% OH do not have a visible influence on the surface topography of the spin-coated films. P(g_4_2T-T)-8% OH APTMS and PEG-COOH-modified films also show similar visual surface morphology (see Figure S2B, middle and right). The surface roughness value of p(g_4_2T-T)-8% OH APTMS (RMS = 2.2 nm) is similar to the pristine surface, and for p(g_4_2T-T)-8% OH PEG-COOH is slightly higher (RMS = 3.8 nm) but still in the low nanometer range, showing that the silanization process has no negative impact on the microstructure of the polymer films.

**Figure 3:**
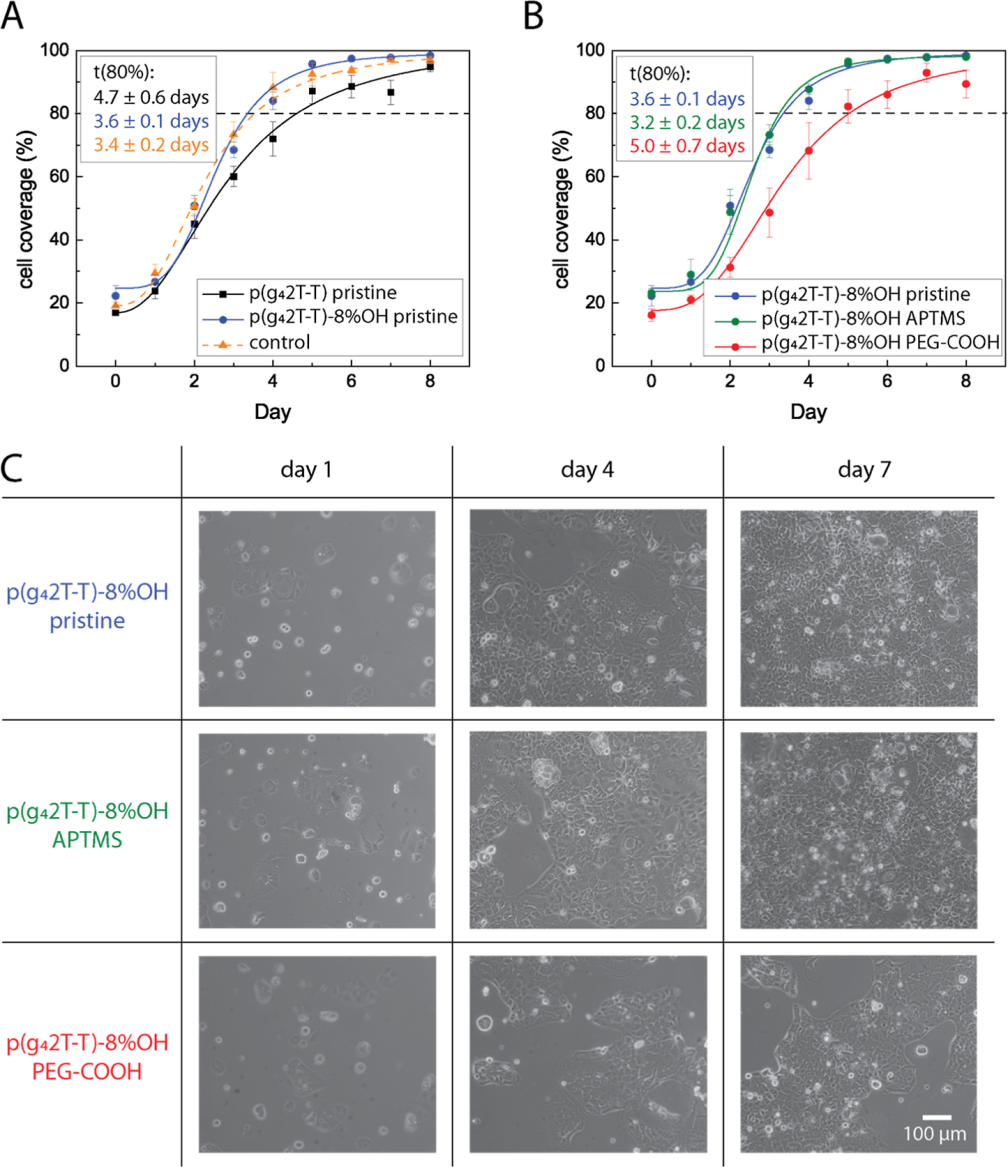
(A) Cell coverage analysis results of Caco-2 cells growing on pristine p(g_4_2T-T)-8% OH, pristine p(g_4_2T-T), and in a standard cell culture well plate as the control. Caco-2 cells grow similarly on pristine p(g_4_2T-T)-8% OH and in control well plates, while inhibited growth is visible on p(g_4_2T-T) (*n* = 4 samples). *t*-test *p*-values for *t*(80%): control vs p(g_4_2T-T)-8% OH *p* = 0.68, control vs p(g_4_2T-T)-8 *p* = 0.02, and p(g_4_2T-T)-8% OH vs p(g_4_2T-T) *p* = 0.06. ANOVA *p* value = 0.03. (B) Cell coverage analysis result of Caco-2 cells on p(g_4_2T-T)-8% OH and p(g_4_2T-T)-8% OH silanized with APTMS or PEG-COOH (*n* = 4 samples) showing inhibited growth in the presence of PEG-COOH. *t*-test *p*-values for *t*(80%): pristine vs APTMS *p* = 0.22, pristine vs PEG-COOH *p* = 0.09, and APTMS vs PEG-COOH *p* = 0.05. ANOVA *p* value = 0.04. Data in (A,B) was fitted to a sigmoidal logistic curve. Error bars show the standard error of the mean. *T*(80%) values were obtained from the fitted curves. (C) Brightfield image series of Caco-2 cells growing on pristine p(g_4_2T-T)-8% OH and p(g_4_2T-T)-8% OH silanized with APTMS or PEG-COOH. Cells growing on PEG-COOH-modified p(g_4_2T-T)-8% OH are hindered in forming a confluent cell layer. Selected images were taken 1, 4, or 7 days after cell seeding. All images are on the same scale.

Next, we evaluated the impact of hydroxyl side chains on the attachment and proliferation of the live cells. To do so, we cultivated Caco-2 cells on p(g_4_2T-T) and p(g_4_2T-T)-8% OH-coated glass coverslips (see Figures 3A and S4). Caco-2 cells are human epithelial cells and are frequently used as a model of the intestinal epithelial barrier as they have the ability to form a confluent and tight cell barrier.^59^ Cell coverage analysis from brightfield images shows that Caco-2 cells grow at a similar rate on p(g_4_2T-T)-8% OH and in conventional cell culture well plate as control, with an 80% confluency reached after 3.6 ± 0.1 and 3.4 ± 0.2 days, respectively (Figures 3A and S4). P(g_4_2T-T) films show a slower growing rate of 4.7 ± 0.6 days, indicating that the presence or absence of the 8% hydroxylated side chains influences cell adhesion on the surface and that the growth of Caco-2 cells is inhibited on p(g_4_2T-T) with respect to pristine p(g_4_2T-T)-8% OH.

We then assessed whether the surface modification with silanes can be used to tune cell interaction with p(g_4_2T-T)-8% OH films. Data for pristine and APTMS-modified p(g_4_2T-T)-8% OH surfaces (see Figure 3B,C) show that a confluency of 80% is reached after 3.6 ± 0.1 and 3.2 ± 0.2 days, respectively. A fully confluent layer is reached approximately 5 days after seeding. In contrast, we observed that cells cultured on PEG-COOH-modified p(g_4_2T-T)-8% OH surface need 5.0 ± 0.7 days to reach a confluency of 80%. Even 8 days after seeding, the cells do not form a fully confluent monolayer. Overall, the results indicate that the presence of additional PEG chains indeed hinders cell adhesion and growth.

As no significant differences could be observed between the pristine and APTMS-modified surfaces using the Caco-2 cells, we further tested the growth of the more sensitive LUHMES cells while differentiating on the pristine and APTMS-modified p(g_4_2T-T)-8% OH (see Figure S5). Brightfield images show that LUHMES cells differentiate normally on the APMTS-modified surface but start to detach and clump up on the pristine p(g_4_2T-T)-8% OH. Thus, the results demonstrate that the silanization step with APMTS improves cell growth for more sensitive cells.

To show that hydroxyl groups, such as those incorporated in p(g_4_2T-T)-8% OH side chains, are needed to enable successful silane functionalization, we tested the adhesion and growth of Caco-2 cells on pristine and silanized p(g_4_2T-T). Brightfield images of p(g_4_2T-T) films silanized with either APTMS or PEG-COOH show similar cell growth coverage of Caco-2 cells with respect to pristine p(g_4_2T-T), with around 4.8–4.9 ± 0.8 days to reach a confluency of 80% (see Figure S6). The data confirm that indeed the absence of hydroxyl side chains prevents surface modification with a simple silanization reaction.

### 3.3 Functionalized Organic Electrochemical Transistors

**Figure 4:**
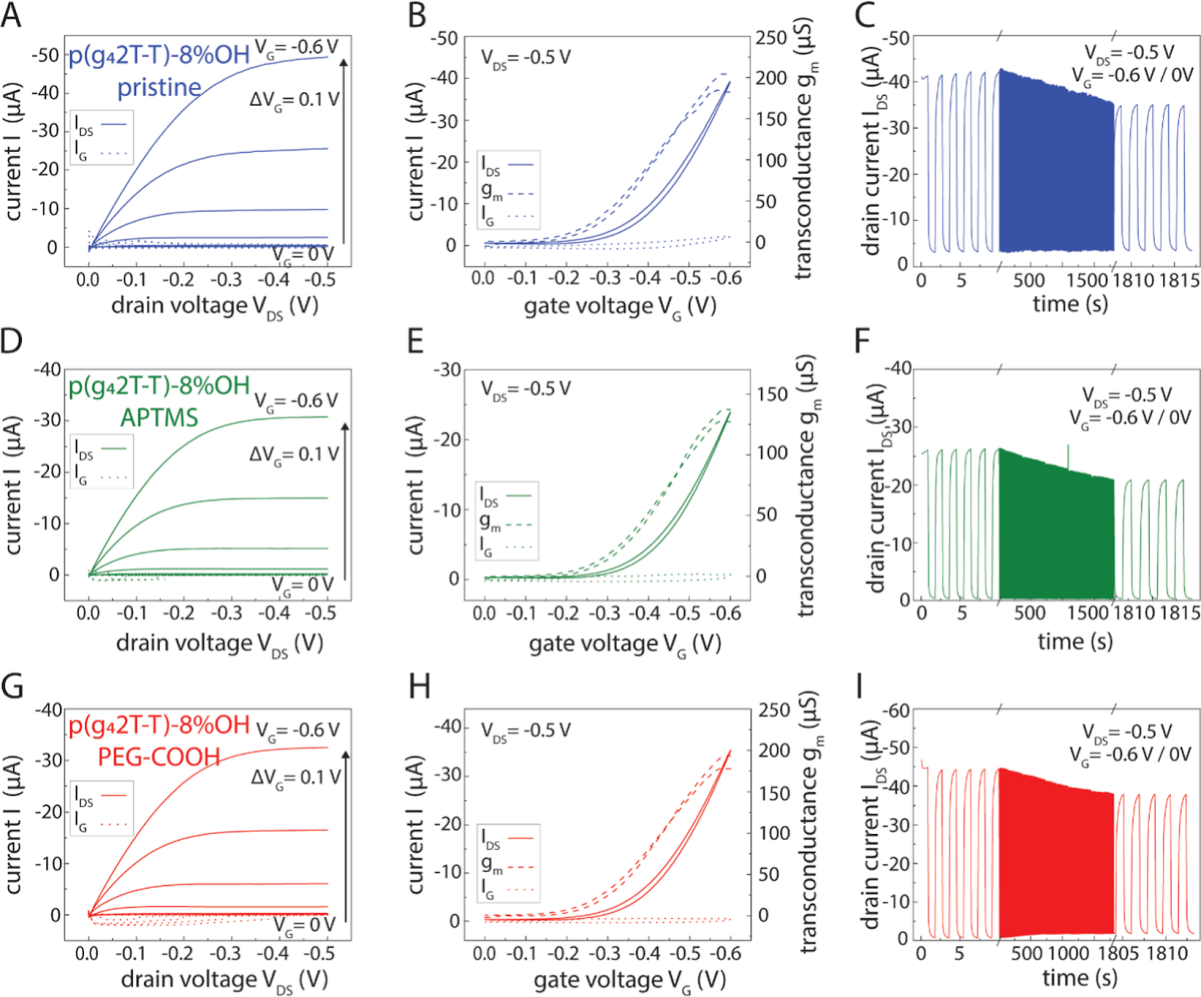
Output, transfer, and switching stability characteristics of OECTs using (A–C) pristine p(g_4_2T-T)-8% OH, (D–F) p(g_4_2T-T)-8% OH silanized with APTMS, and (G–I) p(g_4_2T-T)-8% OH silanized with PEG-COOH. Channel dimensions are *W* = 20 μm and *L* = 200 μm. Ag/AgCl pellet is used as the gate and 0.1 M PBS as the electrolyte. The output and stability switching curves show a representative example measurement, while the transfer curve is an average of 4–6 samples.

As the next step, we fabricated OECTs comprising p(g_4_2T-T) or p(g_4_2T-T)-8% OH to assess the impact of the hydroxyl group-containing monomers on device performance. Output characteristics show that both OECTs made with p(g_4_2T-T) or p(g_4_2T-T)-8% OH operate in accumulation mode at a gate voltage (*V*_G_) between 0 and −0.6 V and a drain voltage (V_D_) between 0 and −0.5 V (Figures 4a and S7a and Table 1). Transfer curves show that p(g_4_2T-T) and p(g_4_2T-T)-8% OH have a similar threshold voltage (*V*_TH_) of −0.22 and −0.23 V and the maximum transconductance at −0.6 V, respectively (Figures 4b and S7b). The on/off current ratio of p(g_4_2T-T), as well as the maximum current, is slightly larger than what is observed for p(g_4_2T-T)-8% OH (see Figure S8 for transfer curves plotted in logarithmic scale). We attribute this difference to the higher average thickness for p(g_4_2T-T) films (68 ± 6 nm) with respect to p(g_4_2T-T)-8% OH films (58 ± 4 nm). The material-related product mobility per volumetric capacitance (μC*) is considered a thickness-independent figure of merit to evaluate mixed ionic-electronic transport properties of the OECT channel material.^60,61^ The value of μC* of 9.5 F/(cm V s) for p(g_4_2T-T) OECTs is comparable to p(g_4_2T-T)-8% OH 9.4 F/(cm V s), indicating that indeed integration of hydroxyl group-containing monomers has no negative impact on the steady-state performance of the OECT. We note that the μC* value for p(g_4_2T-T) is approximately six or 14 times lower than the previously reported values of μC* = 54 F/(cm V s)^41^ and 135 F/(cm V s),^62^ respectively. These differences can be attributed to variations in the OECT fabrication protocols, such as the polymer concentration and solvent used for casting,^63^ the film deposition method (spin coating versus wire bar coating),^64^ the use of postbaking processes,^65^ different electrolyte solutions (10 mM PBS instead of 100 mM NaCl), and variation in the average molecular weight of the polymer.^66^ These differences open up the possibility to further optimize the fabrication process also for p(g_4_2T-T)-8% OH OECTs and ultimately increase the μC*. However, in the scope of this project, we focused on obtaining functional OECTs optimized for cell culture conditions that sustain surface modification treatments when incubated in ethanol-based silane solutions rather than optimizing μC*. For in vitro cell culture sensing applications, the overall OECT performance can further be optimized by varying the channel dimension, for example, increasing the maximum current or transconductance by increasing the thickness and the width or decreasing the length of the channel.

**Table 1:** Summary of the OECT Device Performances with Channel Dimensions of *W* = 200 μm and *L* = 20 μm Using 10 mM PBS as Electrolyte and Ag/AgCl Pellet as the Gate Electrode

polymer	*I*_ON/OFF_	max. *g*_m_ (μS)	max. *g*_m_ norm. (S/cm)	[μC*] (F/(cm V s))	*V*_TH_ (V)	*t*_on_ (ms)	*t*_off_ (ms)
p(g_4_2T-T)-8% OH	130 ± 40	205 ± 43	3.5 ± 0.7	9.4 ± 1.9	–0.23 ± 0.01	539 ± 33	233 ± 20
p(g_4_2T-T)-8% OH APTMS	110 ± 30	137 ± 29	2.4 ± 0.5	7.1 ± 1.5	–0.27 ± 0.01	532 ± 60	216 ± 21
p(g_4_2T-T)-8% OH PEG-COOH	140 ± 20	193 ± 27	3.3 ± 0.5	9.1 ± 1.2	–0.24 ± 0.01	661 ± 79	221 ± 41
p(g_4_2T-T)	150 ± 40	251 ± 52	3.7 ± 0.8	9.5 ± 2.0	–0.22 ± 0.01	745 ± 71	353 ± 49

We further evaluated the operational stability of the OECTs upon on/off cycling. P(g_4_2T-T) shows similar stability to p(g_4_2T-T)-8-% OH, retaining 90 ± 4% and 86 ± 5% of the maximum current after 1000 on/off switching cycles of in ∼30 min with a pulse duration of ∼0.9 s (*n* = 3), respectively (Figures 4c and S7c). The on/off switching times of p(g_4_2T-T) of 745/353 ms (*n* = 6) are slower than p(g_4_2T-T)-8% OH, around 540/230 ms (*n* = 4) (see Table 1 and Figure S9). We attribute this difference to the increased thickness of the spin-coated p(g_4_2T-T) film.^67^ The on switching time can be used to assess growth and barrier formations of cells on an OECT, which is presented in the following chapter. Overall, the data show that integration of hydroxyl group-containing thiophene monomers in the conjugated polymer backbone provides a suitable route to enable further functionalization via silanization without sacrificing device performance.

We then evaluated whether film functionalization with APTMS or PEG-COOH silanes affects p(g_4_2T-T)-8% OH based on the OECT performance. The output, transfer, and switching stability characteristics of pristine p(g_4_2T-T)-8% OH, p(g_4_2T-T)-8% OH APTMS, and p(g_4_2T-T)-8% OH PEG-COOH films are shown in Figure 4. All devices were incubated in ethanol for the same amount of time prior to measurement, as this is a necessary step for both functionalization and sterilization needed for cell culture: the main application of this study. Detailed device parameters are shown in Table 1. Output characteristics show that p(g_4_2T-T)-8% OH APTMS and p(g_4_2T-T)-8% OH PEG-COOH form functional p-type OECTs operating at the same potential as for pristine p(g_4_2T-T)-8% OH OECTs (Figure 4a,d,g). Their on/off current ratios and μC* values derived from the transfer curves vary slightly, but the differences are nonsignificant (ANOVA *p* = 0.72 and *p* = 0.50, see Table 1). Note that we do not expect that surface functionalization with different silanes significantly impacts the thickness of the polymer films. *V*_TH_ values are all within similar ranges of 2.3 and 2.7 V, irrespective of whether the polymer has been functionalized or not (see Table 1), indicating that p(g_4_2T-T)-8% OH can sustain incubation in ethanol-based silane solutions necessary for surface modification and that the silanization process has no negative impact on device steady-state performance. P(g_4_2T-T)-8% OH and p(g_4_2T-T)-8% OH PEG-COOH showed similar operational stability, retaining 86 ± 5% and 89 ± 2% (n = 3) of their maximum current after 1000 on/off switching cycles over a time off ∼30 min with a pulse duration of ∼0.9 s, respectively. p(g_4_2T-T)-8% OH APTMS retained 75 ± 2% (*n* = 3) of its maximum current after 1000 on/off cycles, indicating that functionalization with APTMS leads to slightly lower stability (ANOVA *p* = 0.07). All devices showed comparable on/off switching times of around 600/200 ms (*n* = 6, see Table 1 and Figure S9). Overall, the results demonstrate that the surface modifications performed with ATPMS or PEG-COOH do not significantly impact the performance of p(g_4_2T-T)-8% OH OECTs. Therefore, functionalized p(g_4_2T-T)-8% OH OECTs can be used for specific sensing applications without compromising device performance.

Few published functionalization strategies of conjugated polymers have reported the performance of the OECT device pre- and postfunctionalization. A comparison with the work by Wu, Jiaxin et al.,^34^ where functional COOH groups were introduced by electropolymerizing EDOT and EDOT-COOH, shows that in both approaches, the functionalization does not negatively impact the device performance (see Table S1). However, a detailed comparison remains challenging due to variations in device geometries that affect most of the parameters, apart from the material-related μC*.

### 3.4 Sensing Cell Barrier Formation

**Figure 5:**
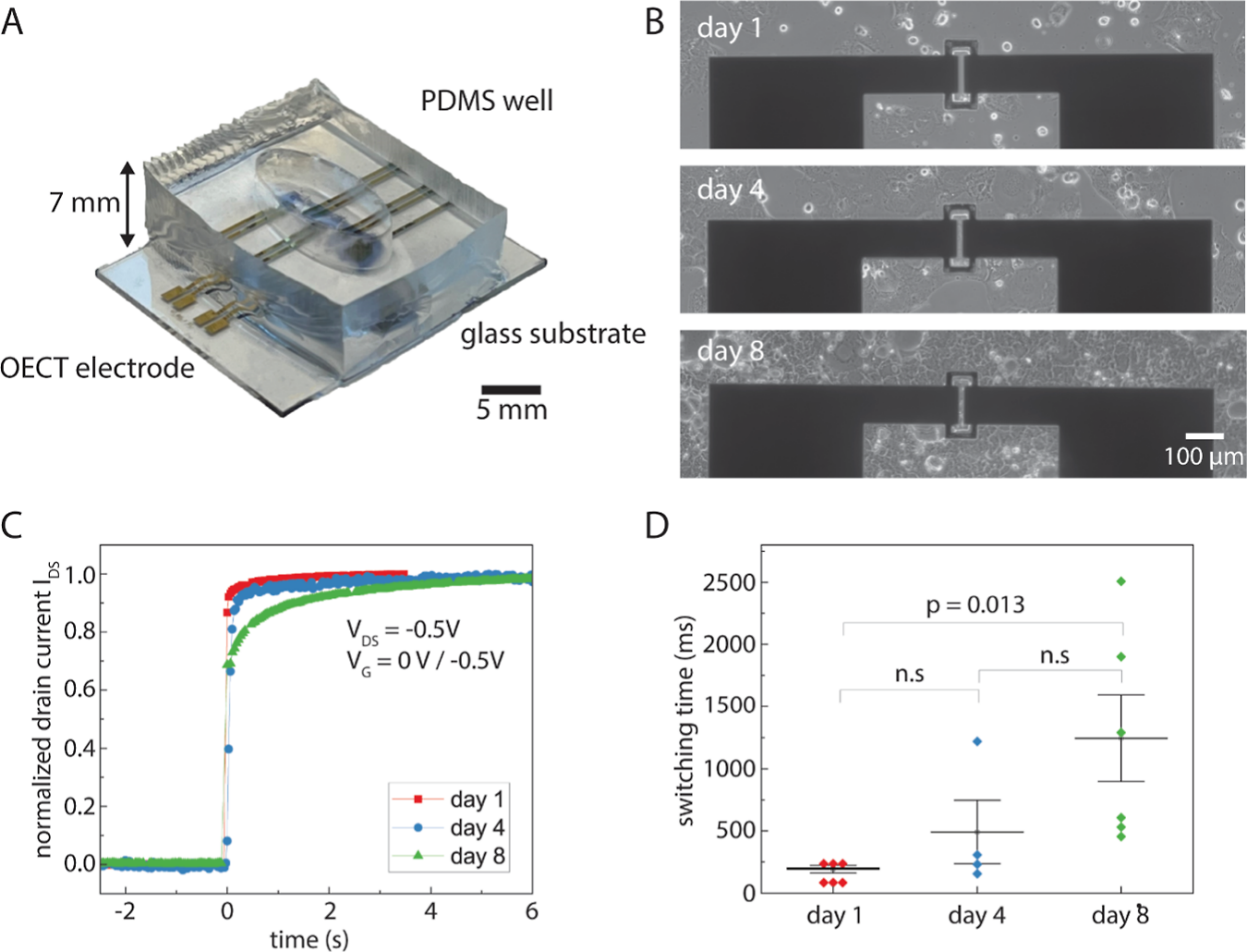
(A) Image of the OECT cell culture device. PDMS well glued on top of the electrode array confines the area where the Caco-2 cells were seeded and cultivated. (B) Time series of bright-field images from Caco-2 cells growing on top of the OECTs. Caco-2 cells grow a confluent and tight layer over the OECT channel within 8 days. (C) Selected representative example of the switching on response of the OECTs in culture with Caco-2 cells. Switching on time increases with an increasing number of days of Caco-2 cell growth. (D) Switching on-times of p(g_4_2T-T)-8% OH OECTs in culture with Caco-2 cells 1, 4, and 8 days after cell seeding. Each point represents the on-time of one OECT for which the on-time of ten consecutive on/off cycles was averaged. Three independent cell culture devices were measured for each measurement day (1, 4, or 8), meaning each device was measured only once. Each cell culture device contained two OECT devices with channel geometry of *L* = 10 μm and *W* = 100 μm. *t*-test was used to calculate the *p* values. Error bars show the standard error of the mean. n.s = not significant.

After establishing the compatibility of p(g_4_2T-T)-8% OH with cells and evaluating the use of hydroxyl groups to enable OECT postfunctionalization, we used p(g_4_2T-T)-8% OH OECTs to assess the cell growth and barrier formation of Caco-2 cells. We designed an OECT cell culture device using a glass substrate with gold source-drain electrodes and polyimide as the insulating layer. OECTs were then patterned using the femtosecond laser-based writing method (see Section 2.8 for details) to obtain well-defined active OECT channel areas with dimensions of *W* = 100 μm and *L* = 10 μm.^10^ Lastly, an oval-shaped PDMS well was glued on top of the substrate to confine the area to cultivate the Caco-2 cells that allows incorporating several OECTs (see Figure 5A). Without the need for any additional coating, the cells grew a confluent layer on top of the OECT layout within 8 days and started to form a barrier expressing the tight junction protein ZO-1 (see Figures 5B and S10). Using a dipped-in Ag/AgCl gate pellet, on/off cycle measurements were performed 1, 4, and 8 days after seeding the cells (Figure 5C). For one on/off cycle measurement, the gate voltage was switched from 0 to −0.5 V for 6 s, and the switching on time was determined.

OECT and optical microscopy data show that the switching on time increases with increasing cell coverage of the Caco-2 cells over time (see Figure 5D). This verifies that the Caco-2 cells started to differentiate and form a cell barrier, reducing the speed of the anions passing through the cell layer. As shown previously for depletion mode OECTs,^23−28^ we could here use the newly introduced semiconducting p(g_4_2T-T)-8% OH polymer to develop the first example of an accumulation-mode OECT to monitor the cell barrier formation in vitro.

## 4 Conclusions

In conclusion, we synthesized the conjugated polymer p(g_4_2T-T)-8% OH, where 8% of the side chains are functionalized with hydroxyl groups. The hydroxyl groups enable facile reactions on the surface of polymer films to modify their properties. We showed that using the two silanes APTMS and PEG-COOH can promote or hinder cell adhesion, respectively. This is the first reported study to show the compatibility of conjugated polymers containing EG side-chains in direct contact with cells for in vitro modeling.

Further, we fabricated OECTs using pristine p(g_4_2T-T)-8% OH as well as p(g_4_2T-T)-8% OH modified with APTMS and PEG-COOH, showing that the surface modification step did not interfere with device operation. Finally, we used p(g_4_2T-T)-8% OH-based OECTs to monitor the cell barrier formation of Caco-2 cells in vitro. In contrast to previously reported PEDOT/PSS-based depletion mode OECTs, we report here the first example of an accumulation mode OECT to monitor cell barrier formation in vitro.

The polymer p(g_4_2T-T)-8% OH can be further used for various sensing approaches in vivo or in vitro. The simple approach of modifying surface properties opens up opportunities to control the interaction with biological materials to facilitate optimal sensing conditions by enhancing, for example, specific cell interactions or hindering unspecific binding. The p-type accumulation mode OECT-based sensing mechanism further expands the toolbox to study the transport of anions through a cell membrane or a lipid bilayer in addition to the cations that can be studied using PEDOT/PSS-based depletion mode OECTs.

## Supporting Information Available

Schematic synthesis overview for p(g_4_2T-T)-8% OH; chemical structure and AFM image of p(g_4_2T-T) and AFM images of p(g_4_2T-T)-8% OH and its silanization with APMTS and PEG-COOH; images of contact angle measurements of p(g_4_2T-T)-8% OH; Caco-2 growth on p(g_4_2T-T) and p(g_4_2T-T); LUHMES growth on p(g_4_2T-T)-8% OH; Caco-2 growth on p(g_4_2T-T); OECT characteristics of p(g_4_2T-T); OECT transfer characteristics in Log scale; OECTs on/off switching curves; fluorescent images of Caco-2 cells on OECTs; detailed description of p(g_4_2T-T)-8% OH synthesis; ^1^H NMR of compound **4**; ^13^C NMR of compound **4**; ^1^H NMR of p(g_4_2T-T)-8% OH; and GPC analysis distribution plot of p(g_4_2T-T)-8% OH (PDF)

## Abbreviations

PEDOT/PSS: poly(3,4-ethylenedioxythiophene)/polystyrene sulfonate
OECT: organic electrochemical transistor
OMIEC: organic mixed ionic-electronic conductor
PEG: polyethylene glycol
APTMS: (3-aminopropyl) trimethoxysilane
PEG-COOH: poly(ethylene glycol)-acetic acid
LUHMES: Lund Human Mesencephalic cells
PLO: poly l-ornithine hydrobromide
DMEM: Dulbecco’s modified Eagle’s medium
FBS: fetal bovine serum
P/S: penicillin/streptomycin
PDMS: polydimethylsiloxane
EG: ethylene glycol
