# Supplementary material for: In Situ Functionalization of Polar Polythiophene-Based Organic Electrochemical Transistor to Interface In Vitro Models
Source: ACS Appl Mater Interfaces. 2024 Sep 27;16(40):54293–304. doi: 10.1021/acsami.4c09197 (PMC11472309; doi:10.1021/acsami.4c09197)
Supplement: Supplementary file 1 — am4c09197_si_001.pdf [file am4c09197_si_001.pdf]

Supporting Information:

In situ functionalization of polar polythiophene-based organic electrochemical transistor to interface *in vitro* models

Sebastian Buchmann[1,2,3], Pepijn Stoop[1,2,3], Kim Roekevisch[1,2,3], Saumey Jain[1,4], Renee Kroon[5], Christian Müller[6], Mahiar M. Hamedi[7,8], Erica Zeglio[2,3,8,9], Anna Herland[1,2,3]*

[1] Division of Nanobiotechnology, Department of Protein Science, SciLifeLab, KTH Royal Institute of Technology, Stockholm, 171 65, Sweden
[2] AIMES – Center for the Advancement of Integrated Medical and Engineering Sciences at Karolinska Institutet and KTH Royal Institute of Technology, Stockholm, 171 65, Sweden
[3] Department of Neuroscience, Karolinska Institutet, 171 77, Stockholm, Sweden
[4] Division of Micro and Nano Systems, Department of Intelligent Systems, KTH Royal Institute of Technology, Stockholm, 100 44, Sweden
[5] Department of Science and Technology, Laboratory of Organic Electronics, Linköping University, Norrköping, 602 21, Sweden
[6] Department of Chemistry and Chemical Engineering, Chalmers University of Technology, Gothenburg, 412 96, Sweden
[7] Division of Fibre Technology, Department of Fibre and Polymer Technology, KTH Royal Institute of Technology, Stockholm, 100 44, Sweden
[8] Digital Futures, Stockholm, 100 44, Sweden
[9] Wallenberg Initiative Materials Science for Sustainability, Department of Materials and Environmental Chemistry, Stockholm University, Stockholm, 106 91, Sweden

* Correspondence: aherland@kth.se

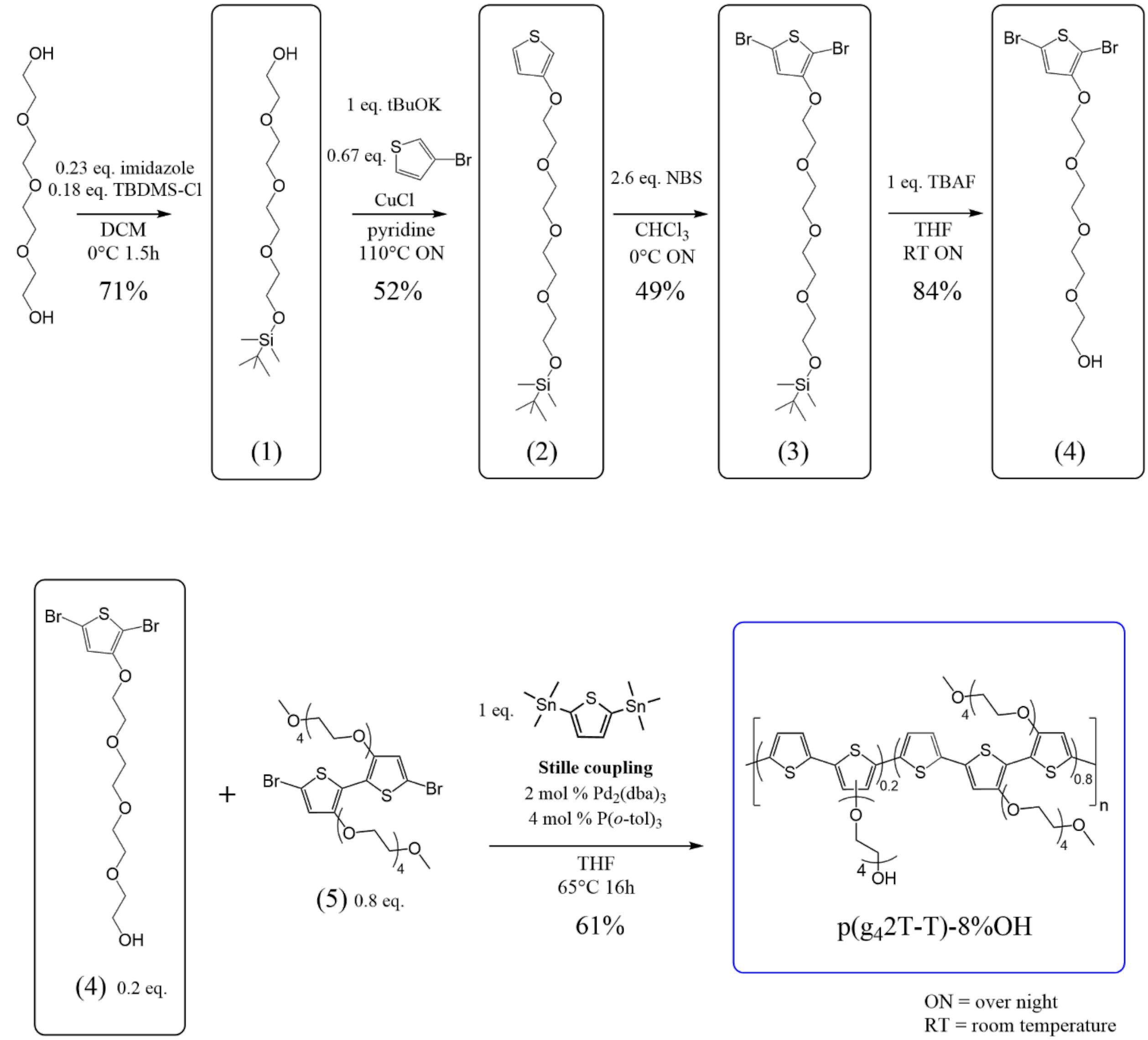

Figure S1: Reaction scheme showing the synthesis step of the conjugated polymer p(g$_4$2T-T)-8%OH, where 8% of the side chains are functionalized with a hydroxyl group.

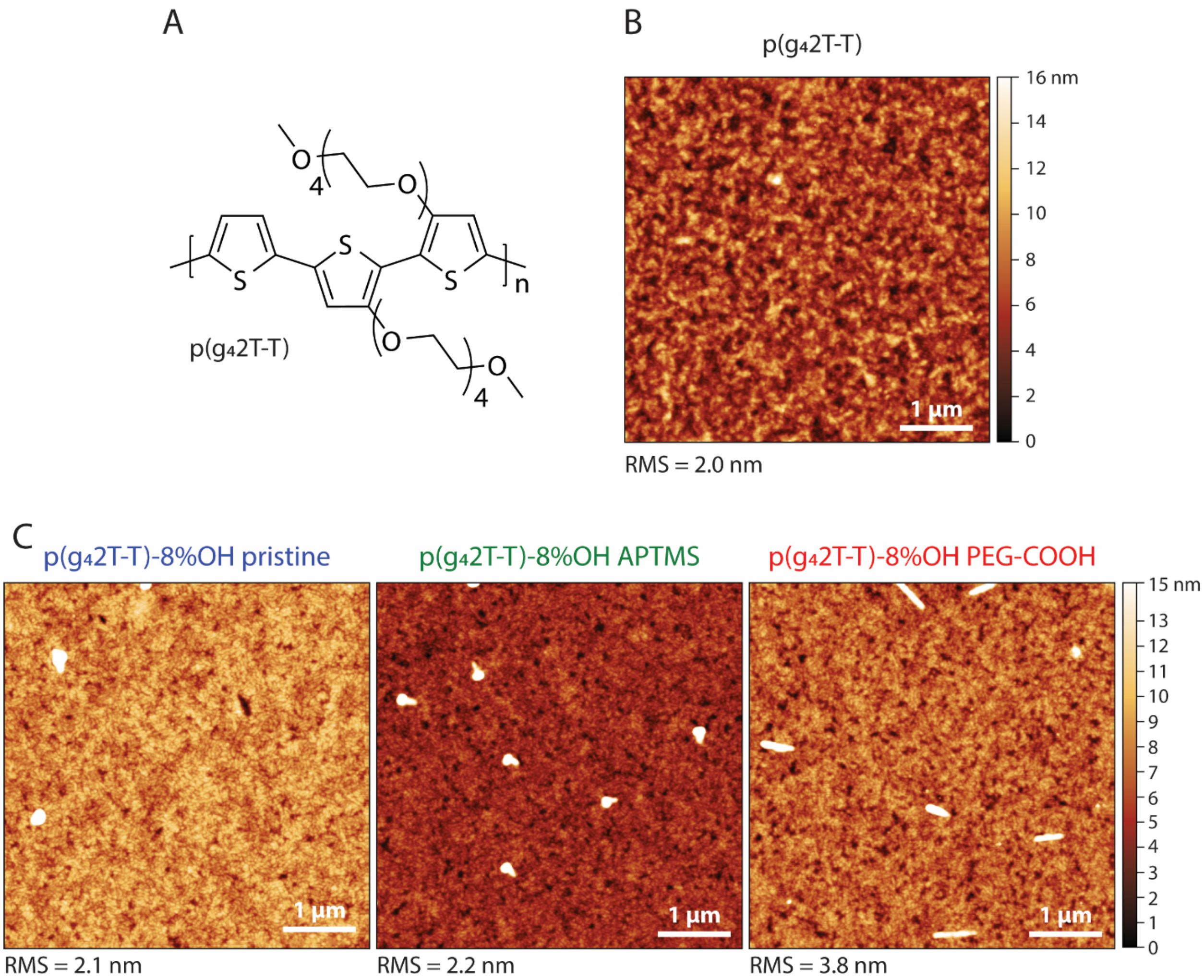

Figure S2: A) Chemical structure of p(g₄2T-T) polymer. B) AFM image of spin-coated dry p(g₄2T-T) film. C) AFM images from spin-coated dry films, pristine p(g₄2T-T)-8%OH (left), p(g₄2T-T)-8%OH APTMS (middle), and p(g₄2T-T)-8%OH PEG-COOH.

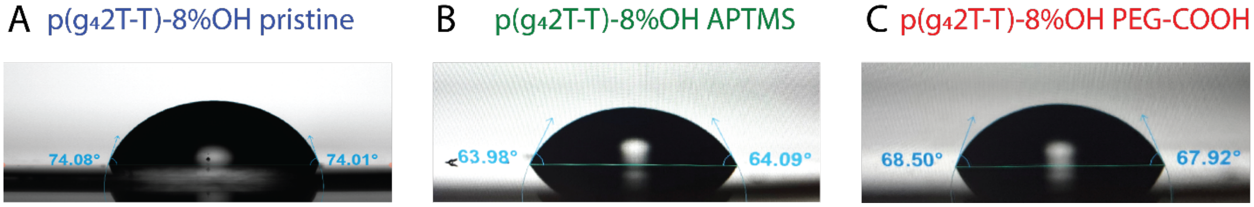

Figure S3: Representative pictures showing the surface contact angle of water on A) pristine p(g₄2T-T)-8%OH, B) p(g₄2T-T)-8%OH silanized with APTMS, and C) p(g₄2T-T)-8%OH silanized with PEG-COOH.

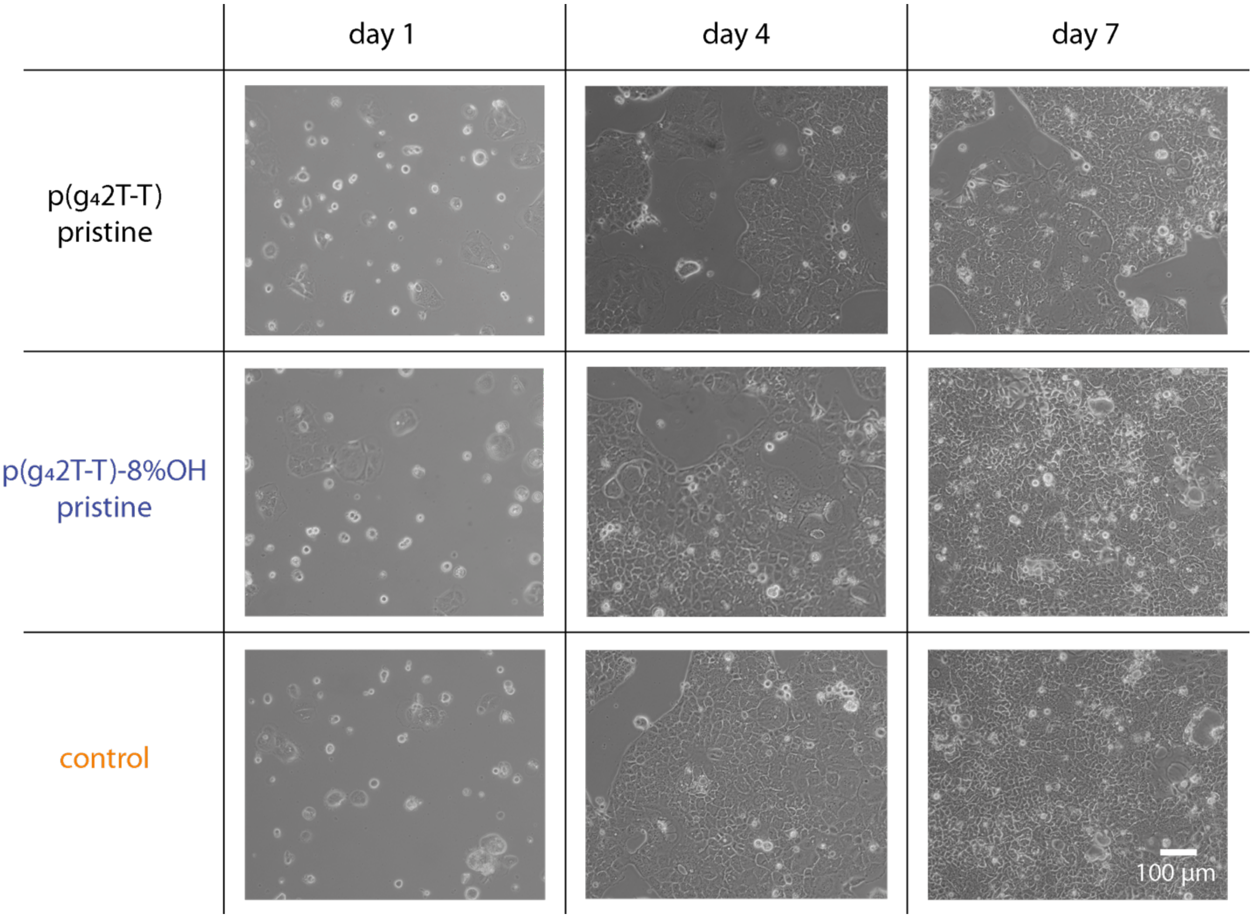

Figure S4: Brightfield image series of Caco-2 cells growing on pristine p($g_4$2T-T), pristine p($g_4$2T-T)-8%OH, and in cell culture well plates as control. Cells growing on p($g_4$2T-T) are hindered in forming a confluent cell layer. The selected images were taken 1, 4, or 7 days after cell seeding. All images are at the same scale.

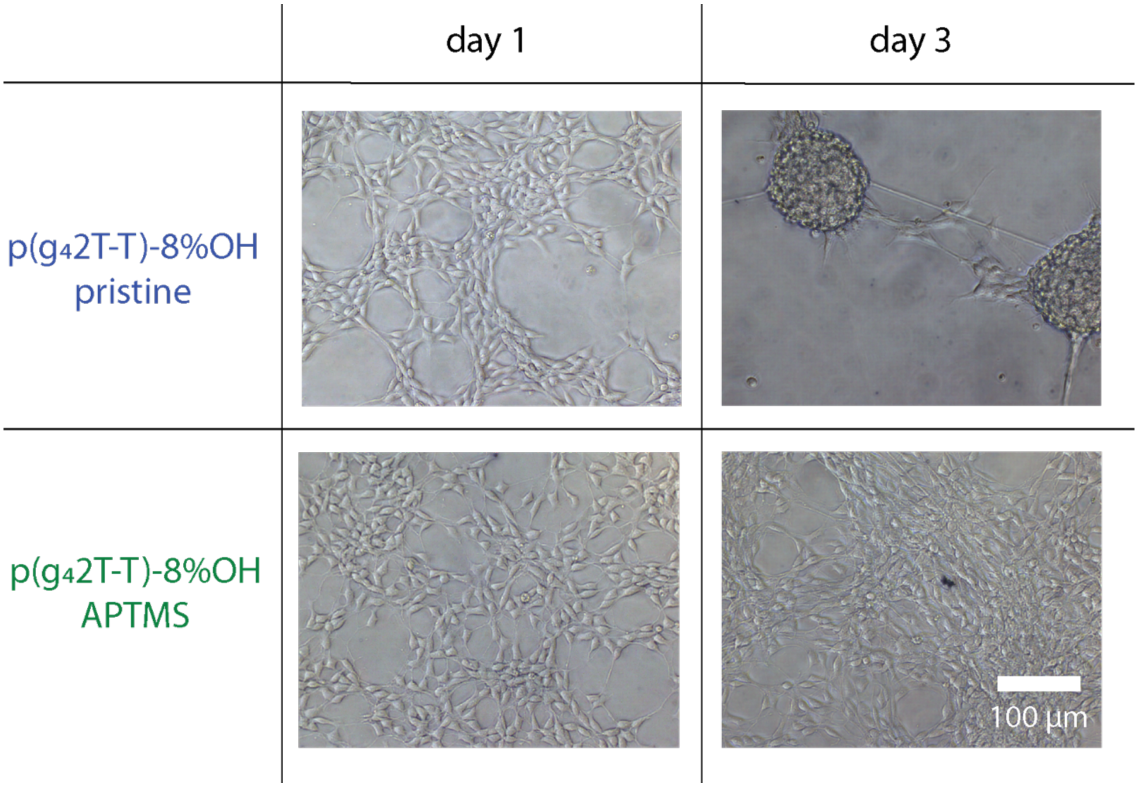

Figure S5: Bright-field images of LUHMES cells differentiating on pristine p($g_4$2T-T)-8%OH and p($g_4$2TT)-8%OH films modified with APTMS one and three days after seeding. All images are at the same scale.

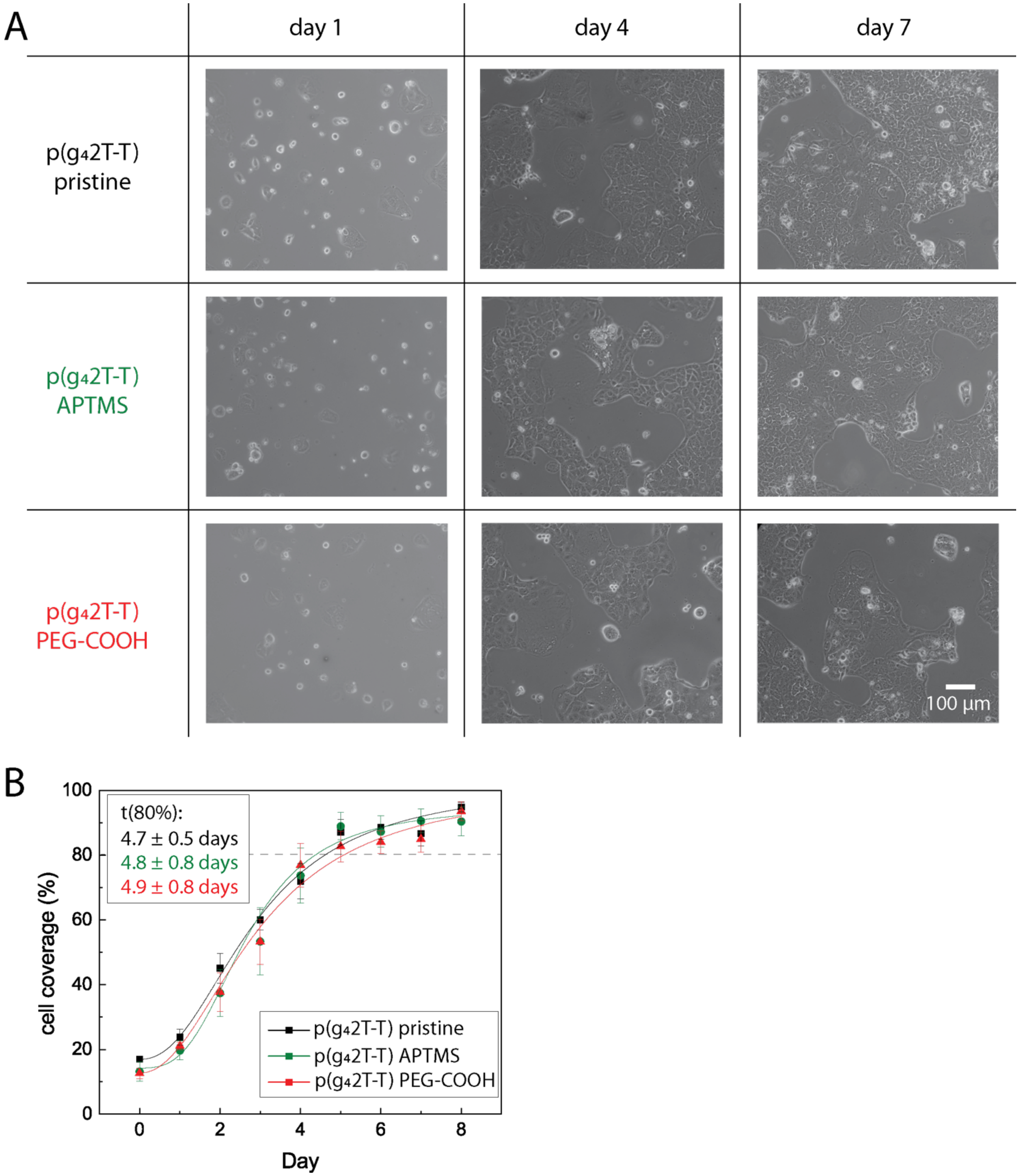

Figure S6: A) Bright-field image series and B) cell coverage analysis of Caco-2 cells growing on p(g$_4$2T-T) and p(g$_4$2T-T) films that were exposed to APTMS or PEG-COOH. No difference in cell growth was observed between the different conditions, indicating that modifications with APTMS or PEG-COOH do not work effectively on the p(g$_4$2T-T), where no hydroxyl groups are present. All images are at the same scale. Data was fitted to a sigmoidal logistic curve. T(80%) values were obtained from the fitted curve and are not significantly different (ANOVA test p=0.99).

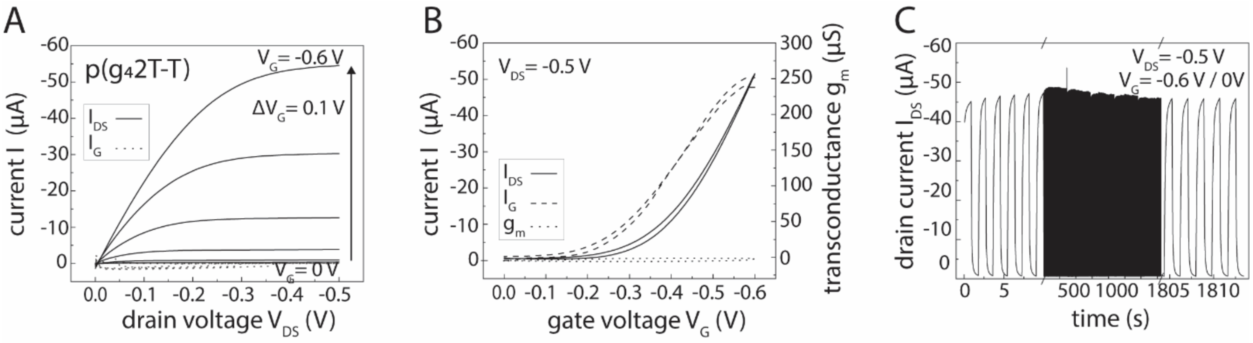

Figure S7: A) Output, B) transfer, and C) switching stability characteristics of p(g₄2T-T) OECTs with channel dimensions of W= 20 μm and L = 200 μm. The output and stability switching curves show a representative example measurement, and the transfer curve is an average of 6 samples.

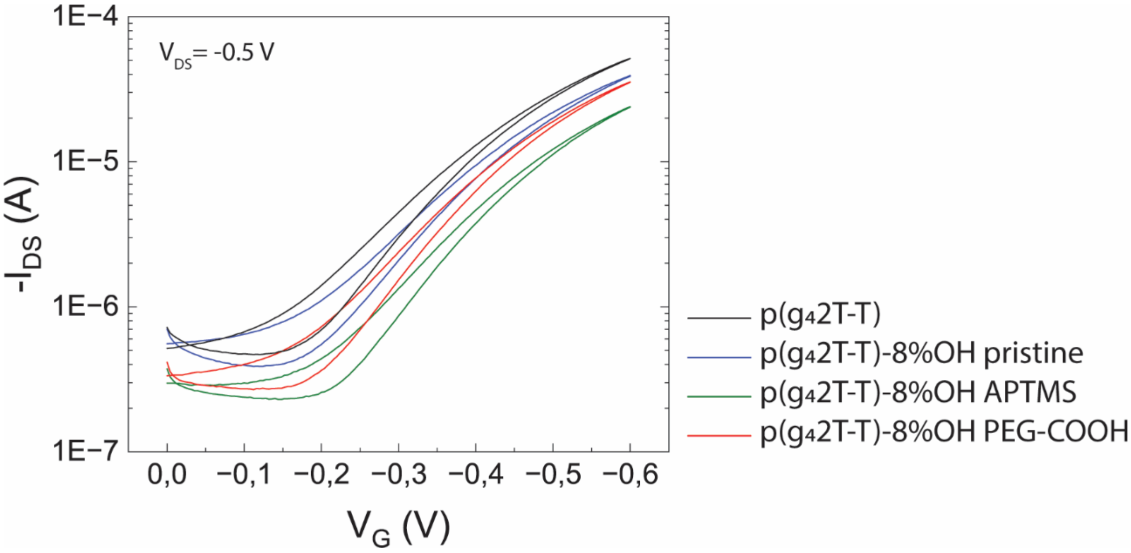

Figure S8: Transfer characteristics in logarithmic scale of OECTs with p(g₄2T-T), p(g₄2T-T)-8%OH, p(g₄2T-T)-8%OH silanized with ATPMS, and p(g₄2T-T)-8%OH silanized with PEG-COOH.

Table S1. Overview comparing pre- and post-functionalized OECT parameters from this work with previously published work by Wu, Jiaxin, et al. [1]

**Polymer**	$I_{ON/OFF}$	**Max. g_m norm. (S/cm)**	**Max. g_m /d. (S/cm)**	**[μC*] (F/(cm V s))**	**V_{TH} (V)**
p(g₄2T-T)-8%OH	130±40	3.5±0.7	35±7	9.4±1.9	-0.23±0.01
p(g₄2T-T)-8%OH APTMS	110±30	2.4±0.5	24±5	7.1±1.5	-0.27±0.01
p(g₄2T-T)-8%OH PEG-COOH	140±20	3.3±0.5	33±5	9.1±1.2	-0.24±0.01
co-PEDOT-2 (EDOTS:EDOTCOOH 7:3 monomer ratio) [1]	1434 ± 592	n.a	114 ± 32	6.35 x 10^{-3}	0.16 ± 0.08
co-PEDOT-2f (functionalized with APMA) [1]	2142 ± 1240	n.a	136 ± 8	10.88 x 10^{-3}	0.09 ± 0.03

APMA =N-(3-Aminopropyl)methacrylamide / n.a = not available.

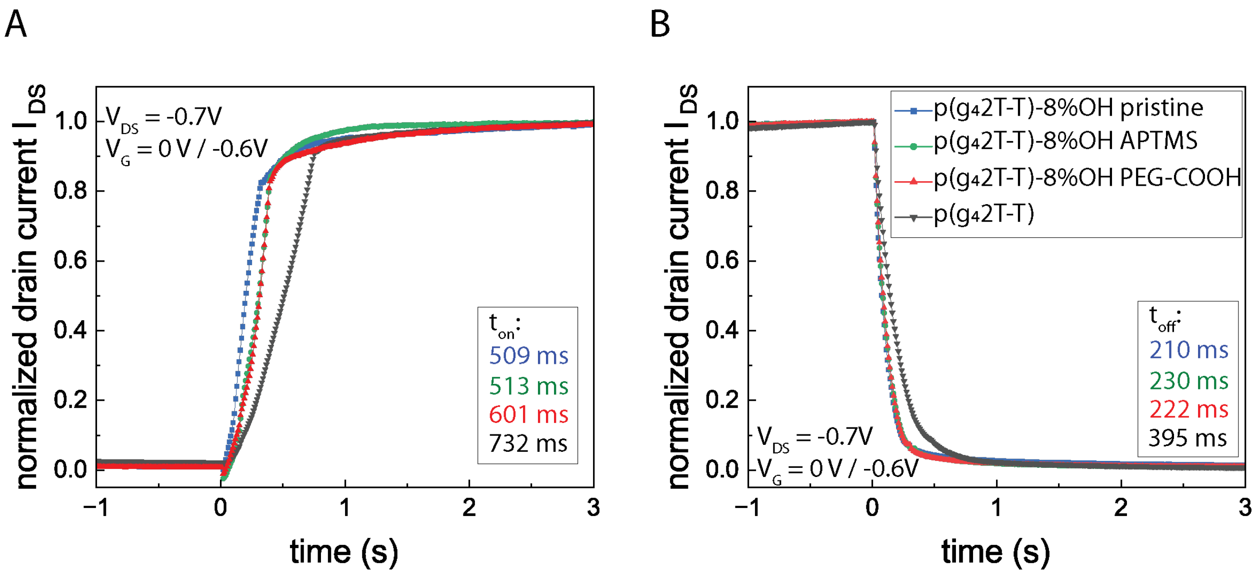

Figure S9: A) Representative example for (A) on and (B) off switching characteristics of p(g₄2T-T)-8%OH, p(g₄2T-T)-8%OH APTMS, p(g₄2T-T)-8%OH PEG-COOH, and p(g₄2T-T) OECTs, respectively.

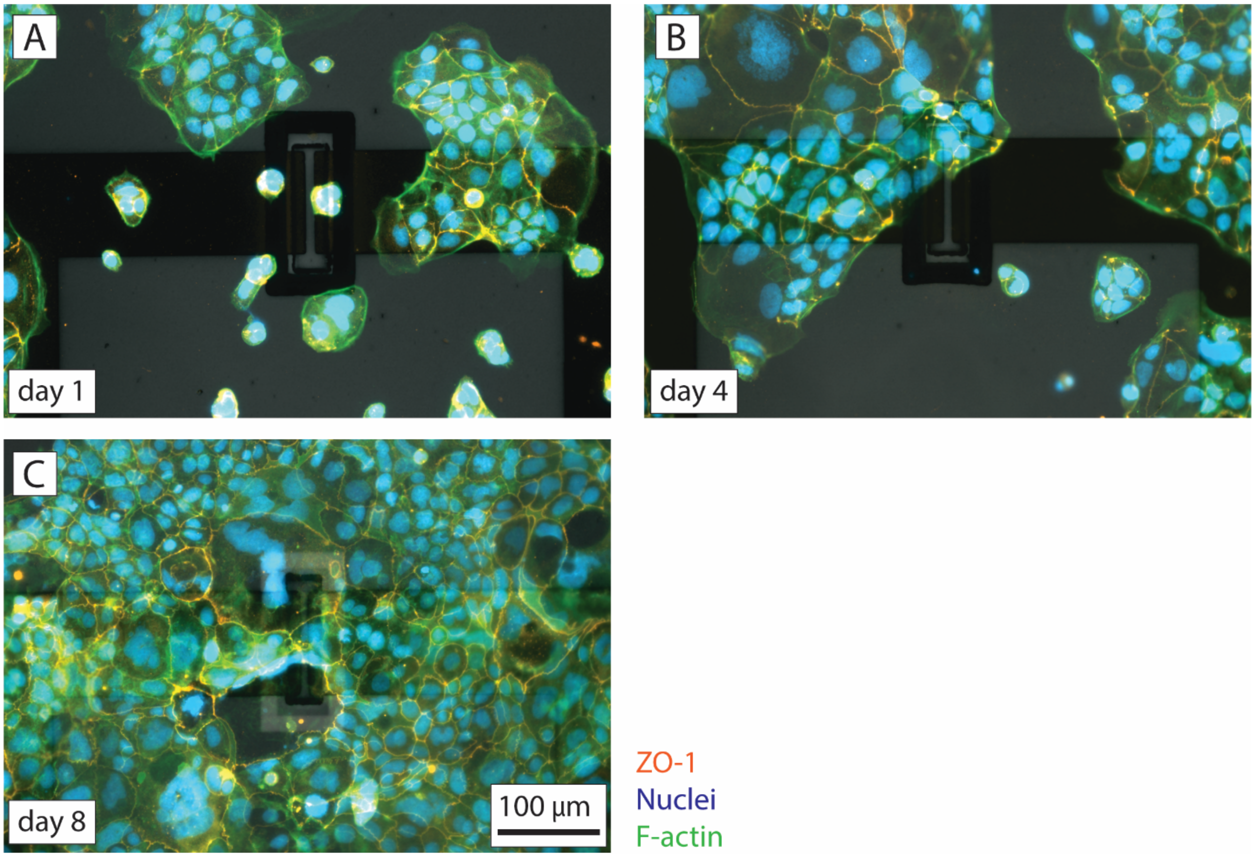

Figure S10: Representative immunocytochemistry fluorescence and bright field images of Caco-2 cells growing on p($g_4$2T-T)-8%OH OECTs A) 1, B) 4, and C) 8 days in culture. Samples are stained with anti-ZO-1 (orange), nuclei/DAPI (blue), and F-actin/phalloidin (green). All images are at the same scale.

Synthesis of p($g_4$2T-T)-8%OH

Compound 1:

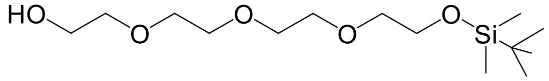

2,2,3,3-tetramethyl-4,7,10,13-tetraoxa-3-silapentadecan-15-ol (1): To a dry one-necked round bottom flask, tetraethylene glycol (15 g, 77 mmol) and imidazole (1.2 g, 17.6 mmol) were added, dissolved into 50 mL DCM, and cooled to 0 °C. A solution of TBDMS-Cl (2.2g, 14.2 mmol) was prepared and dropwise added to tetraethylene glycol solution over the course of 1.5 hours. The reaction was allowed to reach RT while reacting overnight. The reaction mixture was then extracted with DI water (three times), the organic phase dried over $NaSO_4$ and $MgSO_4$, filtered and evaporated to dryness. To remove any aliphatic impurities, the crude product was dissolved in MeOH, extracted with heptane (3 times) and the methanol phase was evaporated to dryness to afford the title compound as a clear oil (3.3 g, 71%). ^{1}H NMR (400 MHz, Chloroform-*d*) δ 3.80 – 3.74 (m, 2H), 3.74 – 3.69 (m, 2H), 3.69 – 3.63 (m, 8H), 3.63 – 3.59 (m, 2H), 3.59 – 3.52 (m, 2H), 2.37 (s, 2H), 0.89 (d, J = 0.7 Hz, 9H), 0.06 (d, J = 0.7 Hz, 6H).^{13}C NMR (101 MHz, Chloroform-*d*) δ 72.64, 72.50, 70.67, 70.33, 62.69, 61.71, 25.91, 18.35, -5.30.

Compound 2:

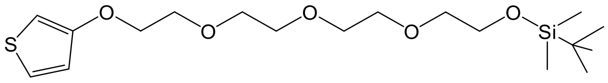

2,2,3,3-tetramethyl-15-(thiophen-3-yloxy)-4,7,10,13-tetraoxa-3-silapentadecane (2): To a dry two-necked roundbottom flask equipped with condenser potassium *tert*-butoxide (1.09 g, 9.7 mmol), copper(I) chloride and anhydrous pyridine (20 mL) were added and stirred. To the resulting suspension compound **1** (3 g, 9.7 mmol) was added dropwise over the course of 5 minutes, and reacted for 1 hour at RT. Then, 3-bromothiophene (1.06 g, 6.5 mmol) was added after which the reaction mixture was heated to 110 °C overnight. The resulting dark brown suspension was cooled to RT, diluted with DCM, filtered and extracted with brine and DI water. The organic phase was dried over $NaSO_4$ and $MgSO_4$, filtered and evaporated to dryness. After column chromatography (DCM + 1% Et_3N) the target compound was obtained as a light-yellow oil (2 g, 53%). ^{1}H NMR (400 MHz, Chloroform-*d*) δ 7.19 – 7.13 (m, 1H), 6.77 (d, J = 5.3, 2.7, 1.4 Hz, 1H), 6.25 (s, 1H), 4.15 – 4.06 (m, 2H), 3.84 (t, J = 4.7, 2.4, 1.1 Hz, 2H), 3.76 (t, 2H), 3.74 – 3.66 (m, 4H), 3.66 – 3.62 (m, 4H), 3.55 (t, J = 5.5, 2.0 Hz, 2H), 0.89 (s, 9H), 0.06 (s, 6H). ^{13}C NMR (101 MHz, Chloroform-*d*) δ 157.58, 124.59, 119.58, 97.49, 72.65, 70.80, 70.78, 70.74, 70.72, 70.69, 70.66, 70.64, 69.67, 69.56, 62.70, 25.92, 18.36, -5.27.

Compound 3

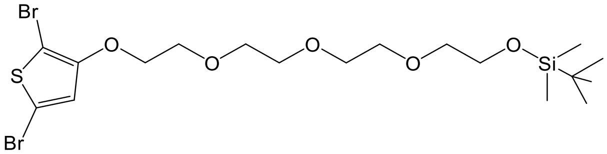

15-((2,5-dibromothiophen-3-yl)oxy)-2,2,3,3-tetramethyl-4,7,10,13-tetraoxa-3-silapentadecane (3): To a dry one-necked roundbottom flask, compound 2 (1.34 g, 3.43 mmol) was dissolved in 50 mL degassed $CHCl_3$ and cooled to 0 °C. Then, NBS (1.22 g, 8.86 mmol) was added in small portions, after which the reaction was allowed to reach RT while reacting overnight. Analysis of the reaction mixture with ^{1}H-NMR showed that only the mono-brominated intermediate had formed. 10 drops of acetic acid were added to the reaction mixture which was stirred for another 4 hours. Then, the reaction was quenched with a saturated solution of $Na_2S_2O_3$, the organic phase extracted with water and brine, dried on Na_2SO_4 and dried *in vacuo*. Gradient column chromatography with DCM + 1% of Et3N and 0à1% MeOH afforded the title compound as a light-yellow oil (900 mg, 49%).^{1}H NMR (400 MHz, Chloroform-*d*) δ 6.81 (s, 1H), 4.18 – 4.13 (m, 2H), 3.81 – 3.74 (m, 4H), 3.73 – 3.63 (m, 8H), 3.55 (t, J = 5.7, 5.1 Hz, 2H), 0.89 (s, 9H), 0.06 (s, 6H). ^{13}C NMR (101 MHz, Chloroform-*d*) δ 153.69, 121.45, 109.56, 91.34, 72.64, 72.01, 70.97, 70.72, 70.69, 70.63, 69.78, 62.69, 25.93, 18.36, -5.27.

Compound 4:

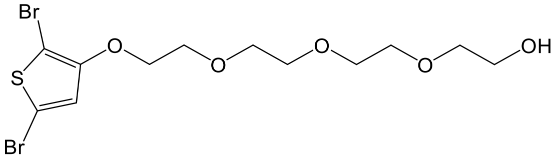

2-(2-(2-(2-((2,5-dibromothiophen-3-yl)oxy)ethoxy)ethoxy)ethoxy)ethan-1-ol (4): In a dry 1-necked roundbottom flask, compound **3** (900 mg, 1.64 mmol) was dissolved in dry THF (20 mL) and TBAF (1 mmol in THF, 1.64 mL) was dropwise added to the stirring solution. The reaction was continued overnight after which it was diluted with ethyl acetate. The organic phase was extracted with water and brine, dried

on Na_sSO_4 and dried *in vacuo*. After column chromatography (1 x DCM, 1 x ethyl acetate) the title compound was isolated as a light-yellow viscous oil (600 mg, 84%).^{1}H NMR (400 MHz, Chloroform-*d*) δ 6.82 (s, 1H), 4.16 (dd, *J* = 5.7, 3.8 Hz, 2H), 3.79 (t, *J* = 5.6, 3.9 Hz, 2H), 3.75 – 3.70 (m, 4H), 3.70 – 3.64 (m, 6H), 3.61 (t, *J* = 5.3, 3.6 Hz, 2H), 2.63 (s, 1H). ^{13}C NMR (101 MHz, Chloroform-*d*) δ 153.68, 121.44, 109.58, 91.31, 72.46, 71.99, 70.95, 70.66, 70.59, 70.34, 69.79, 61.74. HRMS (ESI): calculated: 434.93 (M+H); found: 434.93 (M+H), 456.91 (M+Na).

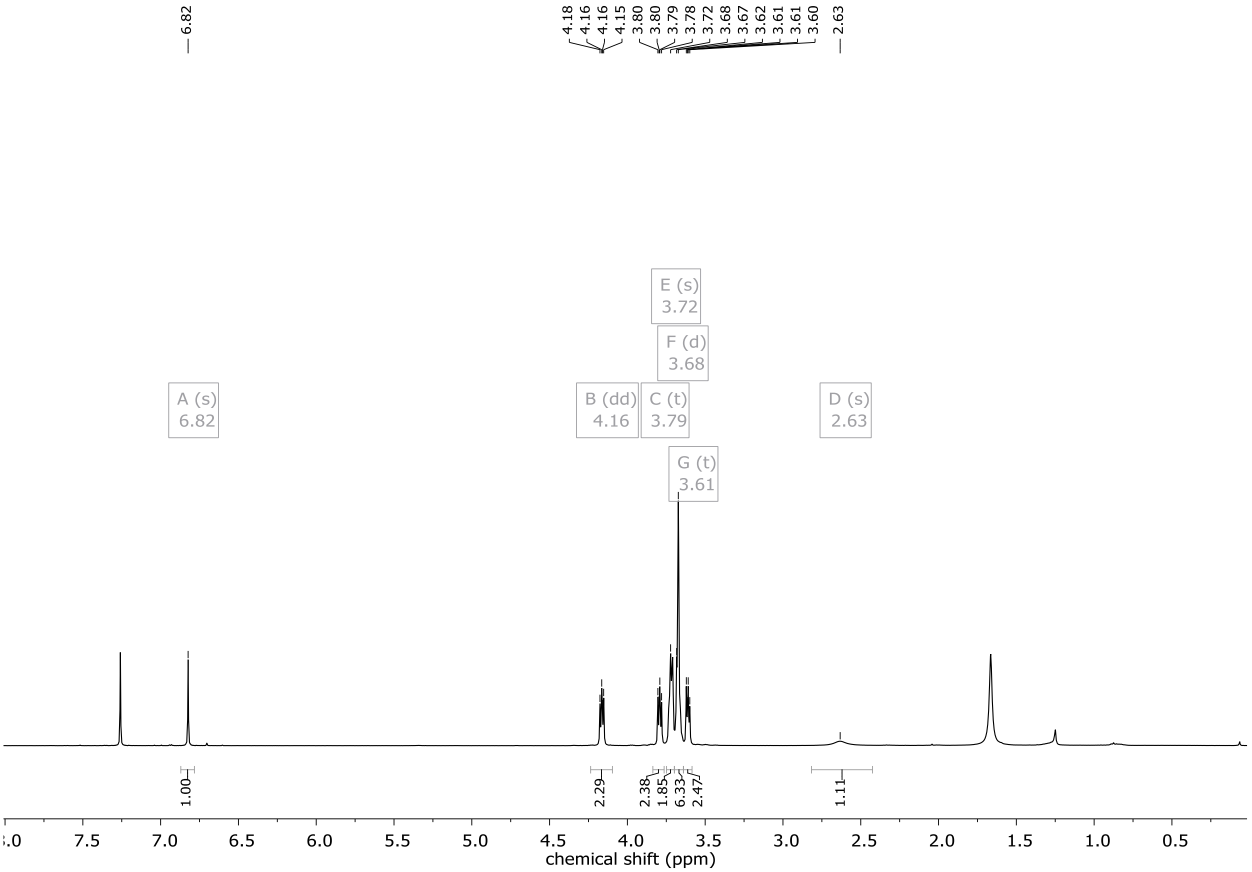

Figure S11: ^{1}H-NMR of compound **4**.

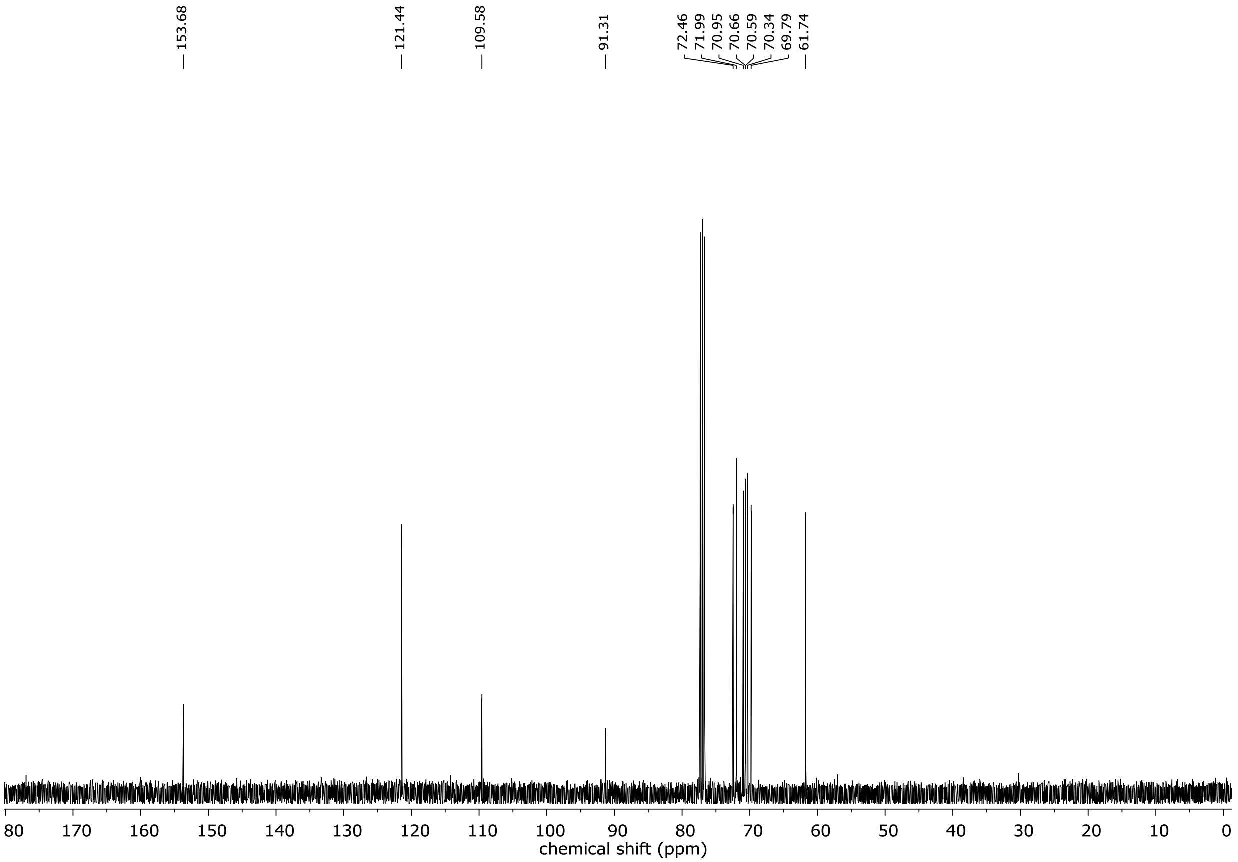

Figure S12: ^{13}C-NMR of compound **4**.

Compound 5: P(g$_4$2T-T)-8%OH

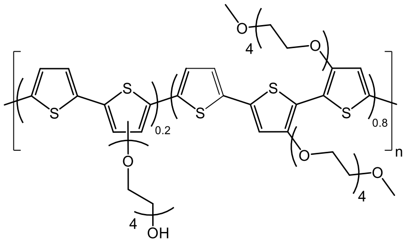

To a dry two-necked roundbottom flask,13,13'-((5,5'-dibromo-[2,2'-bithiophene]-3,3'-diyl)bis(oxy))bis(2,5,8,11-tetraoxatridecane) (compound **5**, 764.1 mg, 1.0374 mmol),[2] 2,5-bis(trimethylstannyl)thiophene (531.37 mg, 1.297 mmol), $Pd_2(dba)_3$ (21.61 mg, 2 mol%) and (*o*-tol)$_3$P (28.7 mg, 4 mol%) were dissolved in THF (20 mL, dry, degassed by N_2 purging for 30 min) and reacted for 10 minutes at 65 °C during which the reaction turned from slightly yellow to deep red. Then, compound **4** (112.6 mg, 0.259 mmol) dissolved in 2 mL of dry degassed THF was added to the reaction mixture which was then continued for 16 hours. The resulting deep purple, viscous solution was precipitated in heptane and filtered which offered the crude polymer as blue sticky fibers. The polymer was dissolved in $CHCl_3$ (100 mL) and vigorously stirred with 100 mL of a saturated sodium diethyldithiocarbamate solution (50 mL) at 60 °C. The mixture was cooled down to RT, the organic phase extensively washed with DI water, concentrated *in vacuo* and precipitated in isopropanol. The polymer was then collected in a thimble and subjected to Soxhlet extraction with isopropanol, diethyl ether and acetone until the extraction solvent was close to colorless. The polymer was then collected by Soxhlet extraction with $CHCl_3$ which was reduced to

approximately 10 mL in volume, precipitated in heptane and filtered onto a 0.45 µm nylon filter, collected and dried *in vacuo* at 40 °C to afford the functionalized polar polythiophene as sticky blue fibers (475 mg, 61% yield).

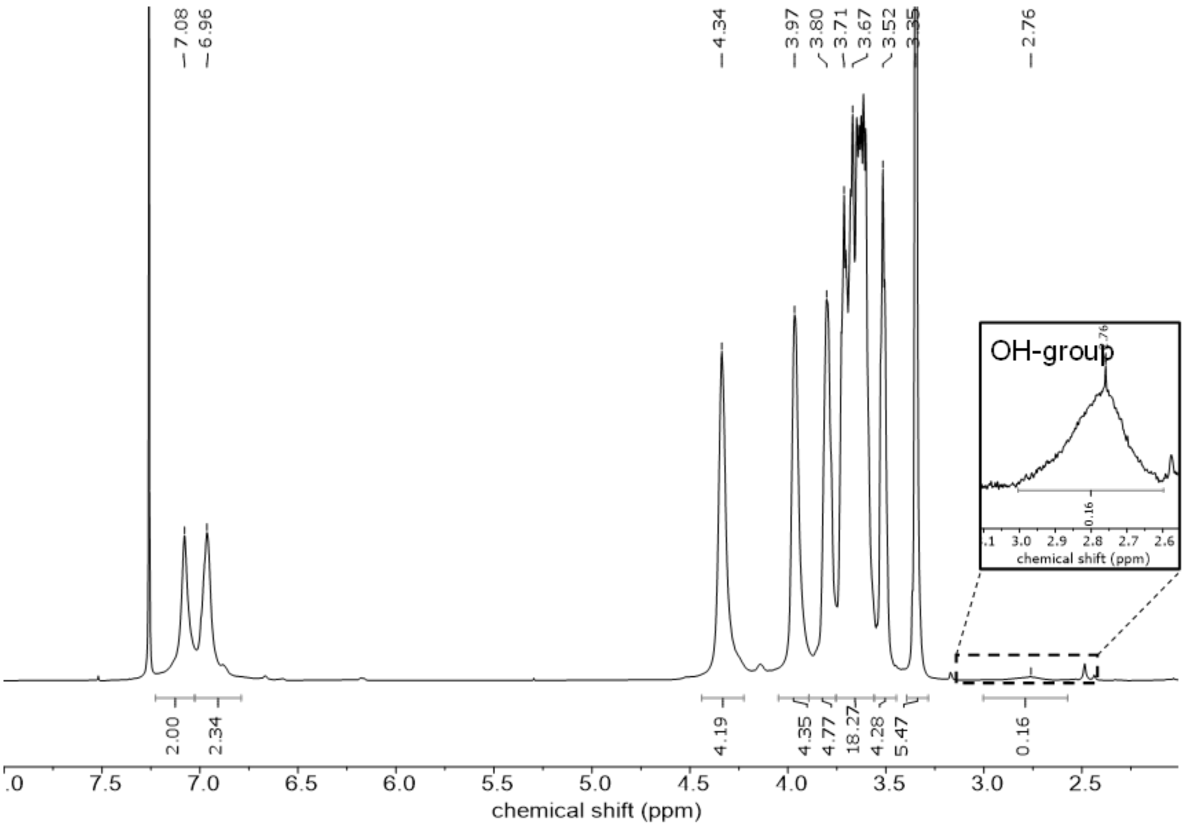

Figure S13: ^{1}H-NMR of p($g_4$2T-T)-8%OH.

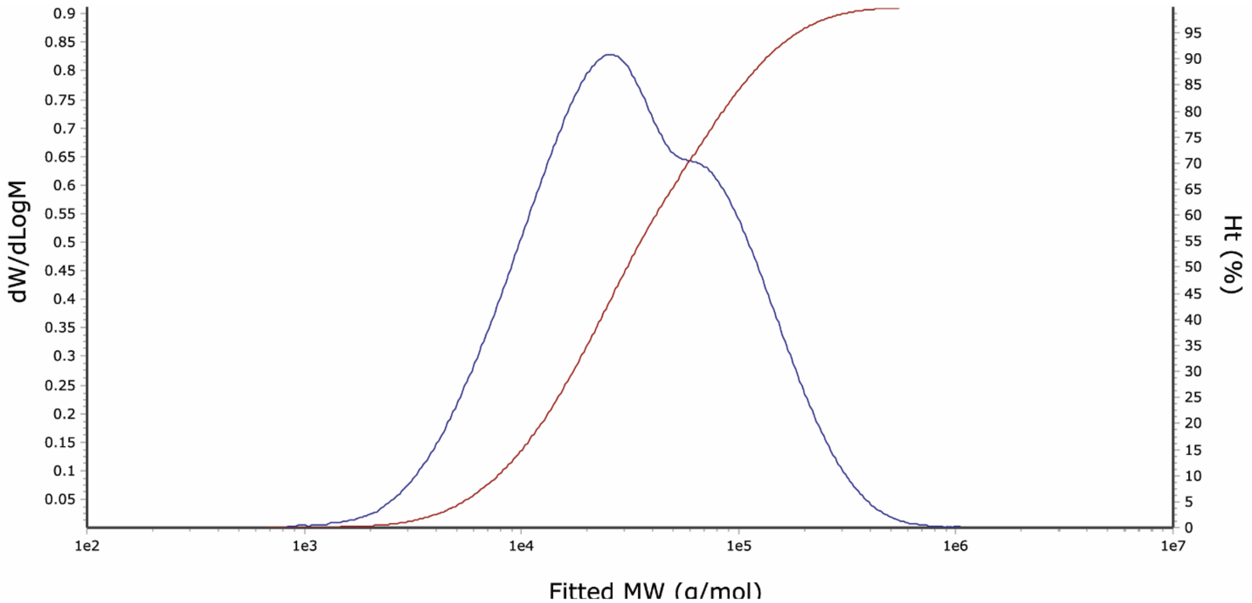

Figure S14: GPC analysis distribution plot of p($g_4$2T-T)-8%OH, having an average molecular weight of Mn 18 kg/mol.

References

[1] J. Wu *et al.*, "Organic Mixed Ionic–Electronic Conductors Based on Tunable and Functional Poly(3,4-ethylenedioxythiophene) Copolymers," *ACS Appl. Mater. Interfaces*, vol. 16, no. 22, pp. 28969–28979, Jun. 2024, doi: 10.1021/acsami.4c03229.

[2] R. Kroon, D. Kiefer, D. Stegerer, L. Yu, M. Sommer, and C. Müller, "Polar Side Chains Enhance Processability, Electrical Conductivity, and Thermal Stability of a Molecularly p-Doped Polythiophene," *Advanced Materials*, vol. 29, no. 24, p. 1700930, 2017, doi: 10.1002/adma.201700930.
